# Cross-sectional study of calves from Norwegian dairy herds with enzootic pneumonia: pathogen occurrence, antimicrobial resistance, culture result interpretation, and sampling site agreement

**DOI:** 10.1186/s12917-026-05557-x

**Published:** 2026-05-18

**Authors:** Lise M. Ånestad, Silje E. Falkeid, Veslemøy S. Oma, Randi T. Garmo, Ane M. Bjelland, Amelia R. Woolums, Maria Stokstad, Thea B. Klem

**Affiliations:** 1 Norwegian Veterinary Institute, Pb. 64, Ås, N-1431 Norway; 2 Department of Production Animal Clinical Sciences, Norwegian University of Life Sciences, Pb. 5003, Ås, 1432 Norway; 3 R&D Department, Farm Advisory Services, TINE SA, BTB-NMBU, Pb. 5003, Ås, 1432 Norway; 4 Department of Bacteriology, Norwegian Institute of Public Health, Pb. 222, Skøyen, Oslo, 0213 Norway; 5 Department of Pathobiology and Population Medicine, Mississippi State University, 240 Wise Center, Mississippi State, Mississippi 39762 USA

**Keywords:** Bovine respiratory disease (BRD), Dairy calves, *Pasteurella multocida*, *Mannheimia haemolytica*, Penicillin, Nasal swab, Nasopharyngeal swab, Bronchoalveolar lavage, Diagnostics, Bacterial abundance

## Abstract

**Background:**

Enzootic pneumonia in calves is associated with pathogens such as *Pasteurellaceae* bacteria and *Mycoplasmopsis bovis*. Some of these bacteria are commensals in healthy calves, complicating culture interpretation, discouraging laboratory diagnostic use, and limiting knowledge of their impact. This study aimed to investigate the occurrence and antimicrobial resistance pattern of respiratory pathogens in calves from dairy herds with enzootic pneumonia. Culture results between upper and lower airway sites in healthy and diseased calves were also compared to evaluate their diagnostic value. A cross-sectional study was conducted on 131 calves (72 healthy and 59 diseased) from nine Norwegian dairy herds. Nasal swabs (NS), nasopharyngeal swabs (NPS), and serum were obtained from each calf, and bronchoalveolar lavage (BAL) from 113 calves.

**Results:**

At the calf level, *Pasteurella multocida* was present in 60%, *Mannheimia haemolytica* in 55%, and *Histophilus somni* in 3%. At the sampling site level, from NS, NPS, and BAL, *P. multocida* was detected in 47%, 45%, and 27%, and *M. haemolytica* in 46%, 37%, and 8%, respectively. *H. somni* was detected in ≤ 2% per site. *P. multocida* appeared as pure culture in 73% (22/30) of positive BAL cultures. Serum antibodies to *M. bovis* were not detected. Most *Pasteurellaceae* isolates were susceptible to penicillin and other common pneumonia antimicrobials (disc diffusion). The exceptions were penicillin-resistant *M. haemolytica* isolates in two calves. Logistic regression identified an association between dominant, abundant cultures of *P. multocida* from BAL and clinical disease. Agreement for *P. multocida* detection between NS and NPS cultures and BAL was fair (kappa). Positive predictive values of *P. multocida* culture results at different abundance levels in NS and NPS, relative to BAL, were at most 49%.

**Conclusion:**

*P. multocida* was the predominant bacterium in lungs of calves from Norwegian dairy herds with enzootic pneumonia, and its abundance in the lungs may help differentiate infection from colonization in clinically diseased calves. *M. haemolytica* and *H. somni* appeared to have less clinical impact. Antimicrobial resistance appeared to be low. Upper airway cultures were inaccurate predictors of bacterial presence and abundance in the lungs and must be interpreted carefully alongside other diagnostic tools.

**Supplementary Information:**

The online version contains supplementary material available at https://doi.org/10.1186/s12917-026-05557-x.

## Background

Respiratory tract infections, commonly known as bovine respiratory disease complex (BRD), is the largest disease problem in calves and young stock of all cattle production systems worldwide [1–3], including Norwegian dairy calves [4]. The disease complex has a major negative impact on animal welfare and economy through increased morbidity and mortality, and lifelong reduced productivity [1–3, 5]. In addition, BRD is the leading cause of antimicrobial (AM) treatment in calves and young stock, and contributes substantially to driving AM resistance (AMR) globally [6, 7]. BRD is challenging to control due to its multifactorial etiology, where pneumonia results from complex interactions between environmental stressors, a suppressed immune system, and multiple viral and bacterial pathogens [8].

Viruses involved in BRD are considered primary pathogens and include bovine respiratory syncytial virus (BRSV; also called bovine orthopneumovirus), bovine coronavirus (BCoV), bovine parainfluenza virus type 3 (BPIV3), bovine herpesvirus type 1 (BHV1), and bovine viral diarrhea virus (BVDV) [9, 10]. However, Norway is declared free from BHV1 and BVDV [11, 12]. Among bacteria associated with BRD, the most important include *Pasteurella multocida*,* Mannheimia haemolytica*, and *Histophilus somni* from the *Pasteurellaceae* family [13]. These are generally considered commensals of the upper airways but can colonize and infect the lower airways, often following a viral infection [8]. The emerging primary pathogen *Mycoplasmopsis bovis* (formerly *Mycoplasma bovis*) has not been detected in Norwegian livestock [14–16], but is an increasing problem worldwide, including in neighboring countries [17–19]. The species was reclassified in 2018 following a whole-genome sequencing-based revision of mycoplasma taxonomy [20]; however, the former name remains widely used. Other less important, but common BRD pathogens are opportunists such as *Trueperella pyogenes* and *Bibersteinia trehalosi* [21].

Clinical BRD is commonly controlled with AMs [6]. In Nordic countries, procaine benzylpenicillin, tetracycline, and florfenicol are among the most commonly used [22, 23]. The widespread use of AMs, including broad-spectrum drugs classified by the European Medicines Agency (EMA) as critically important in human medicine, contributes to the emergence of multi-resistant bacteria [6]. As a result, a trend of decreasing susceptibility of *Pasteurellaceae* spp. to AMs commonly used in BRD treatment, is apparent in multiple countries [6, 24, 25]. A recent study has indicated that penicillin is as effective for treating BRD as the broad-spectrum drugs tetracycline and florfenicol [22]. Despite this, the use of penicillin for BRD treatment is low in most countries, partly due to the need for repeated dosing of commonly used formulations such as procaine benzylpenicillin, the presence of penicillin resistance, and the fact that *M. bovis* is naturally resistant to penicillin [17]. Norway is unique in that penicillin is recommended as first choice treatment for BRD, as reflected in the national AM therapy guidelines [23]. These recommendations rely on the absence of *M. bovis* and on occurring BRD bacteria to be penicillin-susceptible. However, scientific documentation to support these recommendations is limited, as there are no systematic studies of BRD-associated bacteria in Norway.

Data from clinical field samples is also scarce, as most clinicians treat pneumonic calves without the use of laboratory diagnostics. The infrequent use of laboratory analyses may be due to several uncertainties in BRD diagnostics. One challenge is the difficult interpretation of bacteriological culture results. Ideally, a BRD diagnosis should include evidence of lung inflammation or pathology through clinical assessment, transthoracic ultrasonography, or inflammatory cytology from transtracheal aspiration (TTA) or bronchoalveolar lavage (BAL). In addition, microbiological results should identify the causative pathogen and its AMR pattern. However, since BRD-associated bacteria are found in the normal respiratory flora of healthy calves, including in the lower airways, their detection does not necessarily indicate causation [13, 26]. Therefore, many diagnostic laboratories provide quantitative descriptions and the degree of contamination in the culture result, to aid interpretation [27]. Since opportunistic bacteria replicate and adhere more readily when respiratory defenses are impaired, culture results are usually interpreted assuming that larger numbers of bacteria (e.g. dominant or pure cultures) are related to clinical disease [28, 29]. However, among studies that have compared bacterial amount between healthy and diseased calves, a high bacterial amount has not always been consistent with clinical BRD [26, 29, 30].

An additional challenge is choosing the optimal sampling site for identifying BRD pathogens. Commonly used methods include nasal swabs (NS), guarded nasopharyngeal swabs (NPS), and BAL. While upper airway swabs are less invasive than BAL, their accuracy in representing lung pathogens is uncertain and results may be complicated by polymicrobial growth. For BAL, this is less likely and samples are easier to interpret [31]. Agreement studies comparing the upper and lower airway sites in identifying BRD pathogens show variable results [31–36], and thus, there is still no consensus on the best approach [27]. Additionally, if a bacterium is clinically relevant only above a certain amount in the lungs, further research is needed to understand how this is reflected in upper airway sampling sites, as most studies focus only on detection.

To summarize identified knowledge gaps, uncertainties remain regarding the criteria by which presence of *Pasteurellaceae* spp. can be interpreted as clinically relevant, including the importance of bacterial species, amount, and anatomical site. An additional question is whether upper airway sites are reliable proxies for the lungs in identifying respiratory bacteria and their abundance. Norway, with fewer BRD pathogens, provides a favorable setting to study these issues. More knowledge is also needed on the occurrence of bacteria in Norwegian dairy herds with pneumonia and their AMR pattern. This would provide more evidence to current AM therapy guidelines and supplement the limited data currently available in Europe.

Therefore, this study aimed to (1) investigate the occurrence and phenotypic AMR pattern of BRD-associated pathogens in Norwegian dairy herds with enzootic pneumonia, including the frequencies of bacterial co-occurrence and the presence of viral pathogens; and to (2) compare semi-quantitative culture results in the nose, nasopharynx, and lungs of healthy and diseased calves. Our final aim (3) was to determine the diagnostic accuracy of cultures from upper airway sampling sites in identifying bacterial presence and abundance in the lower airway sites, by calculating agreement and predictive values.

## Materials and methods

### Study design

#### Overall design

This cross-sectional study focused on calves in Norwegian dairy herds with enzootic pneumonia problems. The included herds were spread across Southern Norway and visited once at a random time point between March 2021 and February 2022. Laboratory analyses were performed at the Norwegian University of Life Sciences (NMBU) and the Norwegian Veterinary Institute (NVI). The study received ethics approval (ID 26090) from the National Animal Research Authority (part of the Norwegian Food Safety Authority). Participating farms consented to the use of data for research. The individual calf was the study unit. Sample size was determined based on feasibility, while also considering the power needed to detect significant differences. The sample size calculation is described in a separate section.

#### Selection of herds

Inclusion criteria for farms were having a herd size larger than average, i.e. with at least 29.3 cow years (days from first calving to culling within 1 year/365) in 2020 [37], a loose housing system, and at least ten calves diagnosed with BRD per year for the last two years prior to sampling date. The national dairy co-operative TINE provided a list of potential farms. Study herds were selected for convenient access based on geographical location and logistics. We prioritized farms in regions with a high density of dairy herds and selected those that would allow enough time for fieldwork and optimal sample transportation. In potential farms, we also considered the distribution of treatments based on registration data, to avoid those with only short-term acute outbreaks. Eleven farms were invited to participate in the study; seven agreed, while four declined because they no longer had a BRD problem. An additional two farms were recruited through veterinary practitioners, resulting in a total of nine study herds. Samples were collected in March 2021 and between October 2021 and February 2022.

#### Selection of individuals and housing conditions

Calves older than 10 days with and without respiratory disease were included. Exclusion criteria were having received AM treatment or vaccination within 14 days prior to the farm visit or having severe respiratory distress making sedation unsafe. In most herds, the number of calves meeting our selection criteria were limited due to small herd sizes and year-round calving. On the few occasions when more calves met the inclusion criteria than could be sampled during the farm visit, the youngest calves were prioritized for practical reasons. We included as many calves as possible within the time frame of the farm visit. The number of individuals selected per farm varied from 11 to 20 (median = 12), depending on availability and practical concerns.

Selected calves were housed under typical conditions of the Norwegian dairy system: generally, in individual pens during their first 1–3 weeks of life, then moved to larger groups (typically 4–10 calves) in pens with slatted floors, or bedding systems such as deep litter or straw. For calves not weaned, feeding was provided through automatic milk feeders or milk bars with milk replacer or fresh milk, while weaned calves were fed silage and concentrate. Weaning age in the herds was usually 2–3 months.

#### Final study population

Our final study population included 131 calves (68 heifers and 63 bull calves) from nine dairy herds (farm IDs A-I) located in the south-east (*n* = 4), north-east (*n* = 2), south-west (*n* = 1), and north-west (*n* = 2) regions of Southern Norway. Herd sizes ranged from 44.1 to 122.1 cow years (median = 79). Among included calves, there were 109 Norwegian Red, 16 cross breeds, and 6 Holstein. Ages ranged from 11 to 293 days (median = 64), including 91 pre-weaned and 40 post-weaned calves. Twenty-one calves from two herds (farm B and I) had been vaccinated ≥ 39 days prior to sampling. Farm B vaccinated against BRSV and PIV3, both with live agents (*Bovilis RSP Live Vet.*, MSD Animal Health) and Farm I against BRSV, PIV3, and *M. haemolytica*, all with inactivated agents (*Bovilis Bovipast RSP Vet.*, MSD Animal Health).

Further herd- and calf data are summarized in Additional file 1.

### Collection of data and biomaterial

**Fig. 1:**
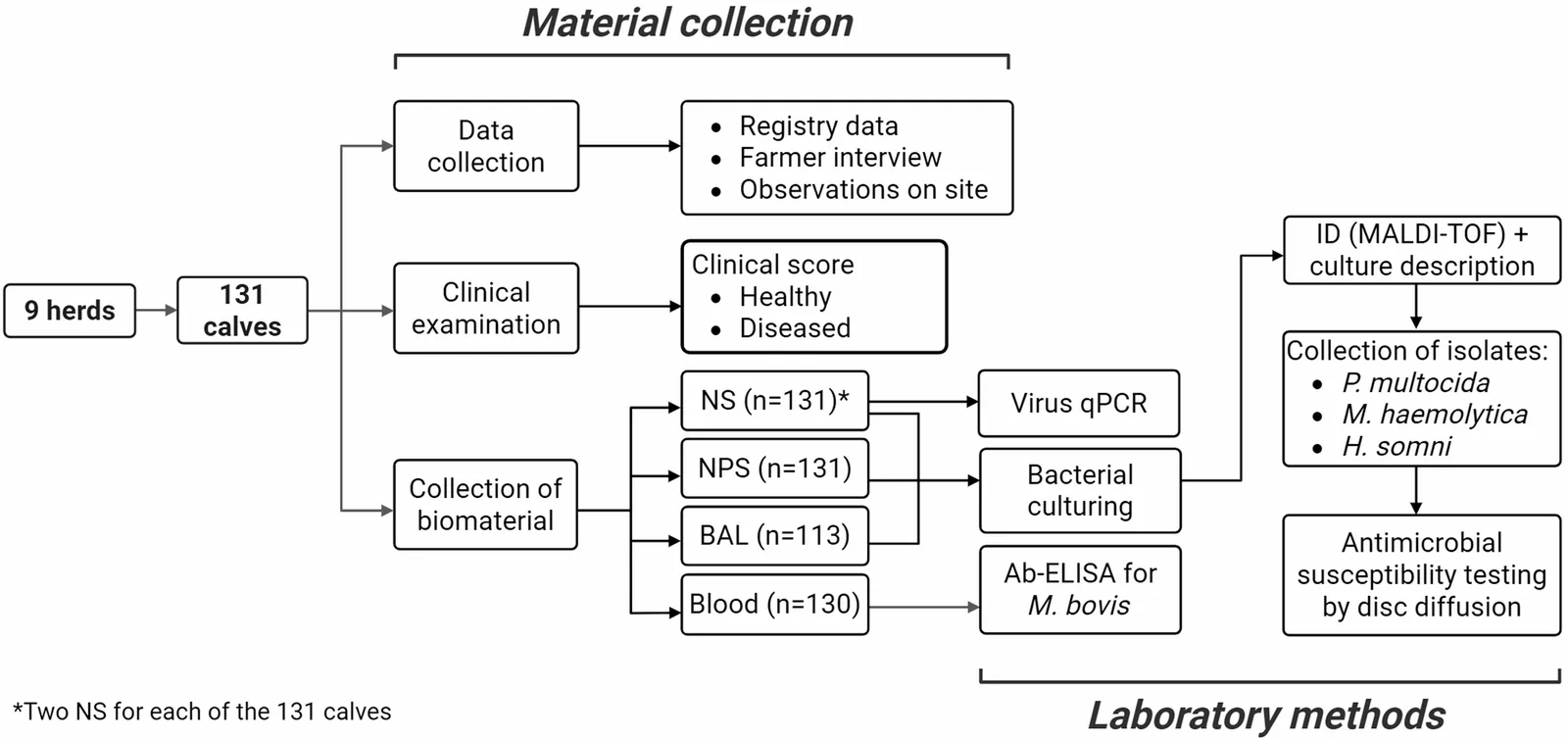
Flow diagram illustrating data and biomaterial collection in the field, and analyses in the laboratory. Two nasal swabs were collected per calf: one for virus qPCR and one for bacterial culturing. For all other sample types, one sample per calf was collected. Abbreviations: NS = nasal swab; NPS = nasopharyngeal swab; BAL = bronchoalveolar lavage. Figure created with BioRender.com

An overview of data and biomaterial collection is presented in Fig. 1.

#### Data collection

We collected registry data, including birth date, gender, breed, and health history, from the Norwegian Dairy Herd Recording System, and data on AM use (drug, treatment date, and length of treatment) from birth until sampling date from the Norwegian Animal Health Recording System. A farmer interview provided additional information about the herd, its health and management history, and AM treatment routines. We also recorded on-site observations, such as housing conditions and feeding routines. The questionnaire forms used are provided in Additional file 2.

#### Clinical examination

First, we observed calves from outside the pen and recorded respiratory rate. Next, we performed the clinical investigations requiring closer contact. In all herds, the same experienced veterinarian performed all the clinical examinations, including thoracic auscultation to avoid inter-rater variability. The exception was 3% (4/132) of the calves, where a second experienced veterinarian assisted. Each clinical parameter was recorded in a clinical scoring system (Table 1) modified from Klem et al. [38]. The system was originally developed for acute viral pneumonia, but adapted by us to also fit chronic BRD. The total clinical score classified calves as healthy or diseased. A calf was classified as diseased if its clinical score was higher than five. The cut-off between the two groups was determined by five experienced veterinarians, focusing on how clinicians would define clinically healthy and diseased calves in a field setting.

**Table 1:** Clinical scoring system of calves

Score^a^	Respiratory rate (breaths/min)	Rectal temperature (°C)	Cough^a^	Nasal discharge	Lung auscultation^b,c^	General appearance^d^
0	≤ 39	≤ 39.5	No cough observed	Normal	Normal	Bright, alert
1	40–55	39.6–39.9	Induced cough	Serous		Mildly depressed
2	56–70	40.0-40.4	1 sporadic cough	Mucous		Moderately depressed
3	> 70	> 40.4	> 1 sporadic cough	Mucopurulent or purulent	Abnormal sounds	Severely depressed

The clinical scoring (CS) system was adapted from Klem et al. Every parameter measured gave up to three points, resulting in a total score of minimum 0 and maximum 18 points. Healthy calves were defined as having a CS of ≤ 5, while diseased calves had a CS of > 5

^a﻿^Cough was determined within the time frame of the individual clinical examination (approximately 10 min)

^b﻿^Auscultation was done in seven different locations of the lung field; dorsal, middle, and ventral on the left and right side, and under the triceps muscle on the right side

^c﻿^Abnormal sounds included crackles, wheezes, and pathologically increased or decreased respiratory sounds

^d﻿^General appearance was evaluated as follows: bright and alert, mildly depressed (standing, calmer than usual), moderately depressed (prefers lying down, stands up in response to contact), or severely depressed (lying down, minimal or no response to contact)

#### Collection and storage of biomaterial

For animal welfare reasons, and in accordance with the ethics approval, all calves were sedated prior to sampling with an intramuscular injection of xylazine 20 mg/ml (1 ml per 100 kg bodyweight). We then sequentially collected NS, guarded NPS, and non-endoscopic BAL fluid, and blood samples from the external jugular vein. Most calves (> 90%) were sampled in sternal recumbency; the rest were standing. First, we wiped the nostrils clean using a single-use woven gauze. Then, we collected one NS (Eswab™490 CE.A, Copan, Brescia, Italy) for bacterial culture and another (Floqswabs^®^502CS01, Copan, Brescia, Italy) for viral qPCR. Both were nylon flocked swabs, which we inserted approximately five cm into the nostril, rotated against the mucosa, and retracted. Next, we collected one NPS (Laryngeal swab™MW128, Medical Wire, Wilts, England) for bacterial culture. We inserted the 27 cm guarded rayon swab into the ventral meatus of the nose to a level 3–4 cm rostral to the eye’s medial canthus. Then, we advanced the inner swab 3–4 cm caudally, rotated it 30 s, and retracted it back into the sheath before withdrawal. The nostril was wiped again, and we inserted a local anesthetic (1 ml procaine hydrochloride 40 mg/ml + adrenaline 0.036 mg/ml diluted with 1 ml sterile saline 0.9%). A sterilized, homemade Teflon BAL catheter (1.5 m; inner and outer diameters of 2 mm and 4 mm, respectively; VWR, Leuven, Belgium), attached to a 12 G steel catheter, was used to collect BAL fluid [31]. With the calf’s neck extended, the BAL catheter was passed through the nose and into a bronchus, confirmed by a wedged tube position. Then, 60 ml of sterile saline 0.9% was injected and aspirated, returning with a distinctive foam layer. Unsuccessful attempts (no fluid or non-foamy) were followed by a second attempt of 60 ml saline.

NS, NPS, and BAL samples for bacterial isolation were stored at 4 °C from sampling until analysis. The swabs were kept in liquid Amies medium without charcoal. NS samples for viral qPCR were put in empty sterile microcentrifuge tubes, snap frozen on dry ice on-site, and then stored at − 80 °C within 24–48 h. Blood samples were collected in plain tubes at room temperature for 1–3 h and then stored at 4 °C until centrifugation. Sera were stored at − 80 °C until analysis.

### Laboratory analyses and handling of samples

#### Bacterial isolation

All samples were cultured within 24 h of collection at NMBU. Material from NS, NPS, and BAL were inoculated onto 5% bovine blood agar (produced at NVI, Ås, Norway). Swabs were directly applied and spread into three dilutions using disposable loops, then streaked through with *Staphylococcus pseudintermedius* (source of V-factor), according to standard methods. Five ml of BAL fluid was vortexed for 5 s, then 1 µl fluid was inoculated onto the agar and plated as before, and 1 ml was added to 5 ml brain heart infusion (BHI) broth. Blood agar plates from swabs and BAL were incubated at 37 °C with 5% CO_2_ for 20–24 h, while BAL enriched with BHI broth was incubated for 20–24 h at 35 °C without added CO_2_. If BAL cultures showed no growth, 1 µl of enriched BAL fluid was cultured onto blood agar and incubated as described for swabs and BAL. Any plates with poor growth were re-incubated for another 20–24 h. Following incubation, we first described the presence, semi-quantitative growth, and presumptive species based on phenotypic traits. For *P. multocida*,* M. haemolytica*, and *H. somni* (collectively referred to as *Pasteurellaceae* spp. in this study), the phenotypic criteria were typical colony morphology and odor. The same investigator described all the plates. From each sampling site, we selected one representative colony per species per site. Presumptive colonies of *P. multocida*,* M. haemolytica*, and *H. somni*, were subcultured on 5% bovine blood agar and incubated for 20–24 h (20–72 h for *H. somni*). We then confirmed species identity of all colonies using matrix-assisted laser desorption/ionization time of flight mass spectrometry (MALDI-TOF MS) with the VITEK^®^MS instrument (bioMérieux, Craponne, France), following the manufacturer’s protocol. The spectra were analyzed using the VITEK^®^MS V3.2 database. For other BRD-associated bacteria, such as *T. pyogenes*, and for fungi, we confirmed the ID of up to three representative colonies per herd and considered morphologically identical colonies within the same herd to be the same species. Fungal colonies were sent to NVI and identified by macro- and micromorphology. All purified isolates of *Pasteurellaceae* spp. were susceptibility tested.

#### Antimicrobial susceptibility analysis

Verified isolates of *P. multocida*,* M. haemolytica*, and *H. somni* were susceptibility tested with the disc diffusion method, following the standard protocol of the European Committee on Antimicrobial Susceptibility Testing (EUCAST). We inoculated the bacteria onto Mueller-Hinton agar plates with 5% citrated bovine blood (produced at NVI, Ås, Norway) and added AMs commonly used in calves. The panel consisted of penicillin (PEN), 10 µg; amoxicillin-clavulanic acid (AMC), 30 µg; trimethoprim + sulfamethoxazole (STX), 1.25/23.75 µg; tetracycline (TET), 30 µg; enrofloxacin (ENR), 5 µg (Rosco Diagnostica, Taastrup, Denmark); florfenicol (FFC), 30 µg; and streptomycin (STREP), 10 µg (Thermo Fisher Scientific, Basingstoke, UK). We incubated the plates for 20–24 h at 35 °C before recording the inhibition zone diameters. Breakpoints applied are described in Additional file 3. We used those of EUCAST where these were available (PEN, AMC, STX, TET) and those of the Clinical and Laboratory Standards Institute (CLSI) otherwise (ENR, FFC, STREP). We reported the values as either susceptible (S), susceptible at increased exposure (I), or resistant (R), according to the criteria described in EUCAST and CLSI guidelines.

In a supplementary study including a subset of calves (*n* = 9) from 6 herds, we collected and susceptibility tested an additional 1–3 isolates per site to compare phenotypic AMR pattern of *P. multocida* within and between sampling sites of the same animal. These results are described separately.

#### Virus detection by real-time PCR

NS samples for viral screening were analyzed at NMBU 1.5-2 years after collection. We performed the RNA extraction using the bioMérieux NucliSens^®^, miniMAG™ system according to the instructions of the manufacturer. We then analyzed the samples for the presence of viruses by real-time PCR (qPCR) using the multiplex Pneumo 4BV kit (DNA diagnostic A/S, Risskov, Denmark) according to manufacturer’s protocol. For viruses, the kit included primers to detect BPIV3, BCoV, BRSV, BVDV, and BHV1. Viruses with a cycle threshold (Ct) value < 37 were considered positive, as recommended by the manufacturer.

#### Detection of antibodies to *Mycoplasmopsis bovis*

Serum samples were analyzed at NVI 1.5-2 years after collection. We used an indirect ELISA kit (IDScreen^®^Mycoplasma bovis Indirect, IDvet, Grabels, France) to investigate if antibodies against *M. bovis* were present, following the manufacturer’s protocol. Each sample was tested in duplicate. The amount of antibodies to *M. bovis* was determined by the sample to positive ratio percentage (S/P %) and labelled positive if the S/P % was ≥ 60%. In case of doubtful or positive reactors, we retested the samples using the same ELISA kit. For negative reactors, we interpreted the samples as negative.

### Data management and statistical analyses

#### Categorization and data management

**Fig. 2:**
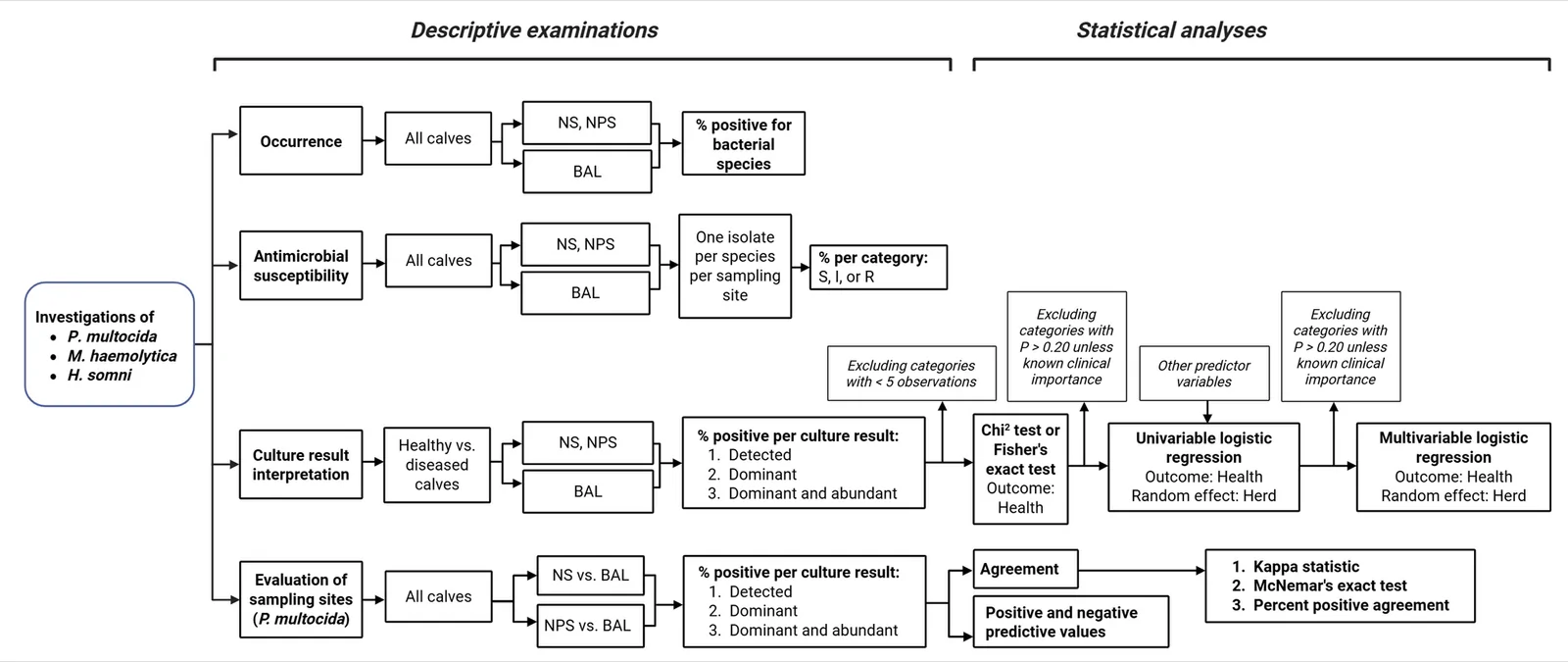
Overview of data analyses for *Pasteurellaceae* species. The figure shows the levels at which analyses were conducted (e.g. in all calves or at group level). NS and NPS represent the upper airway sites (each analyzed separately), while BAL represents the lower airways. For culture result interpretation analyses, each category (e.g. dominant cultures) was assessed per sampling site. Variables with a total of < 5 observations among all calves were not tested. Pearson’s chi-squared test was used to test variables with ≥ 5 observations per health group, while Fisher’s exact test was used if at least one group had < 5 observations. For agreement calculations, only culture results at the detection level were examined, while for predictive values, all three categories of amount were investigated. Abbreviations: NS = nasal swab; NPS = nasopharyngeal swab; BAL = bronchoalveolar lavage; S = susceptible; I = susceptible, increased exposure; R = resistant. Figure created with BioRender.com

**Fig. 3:**
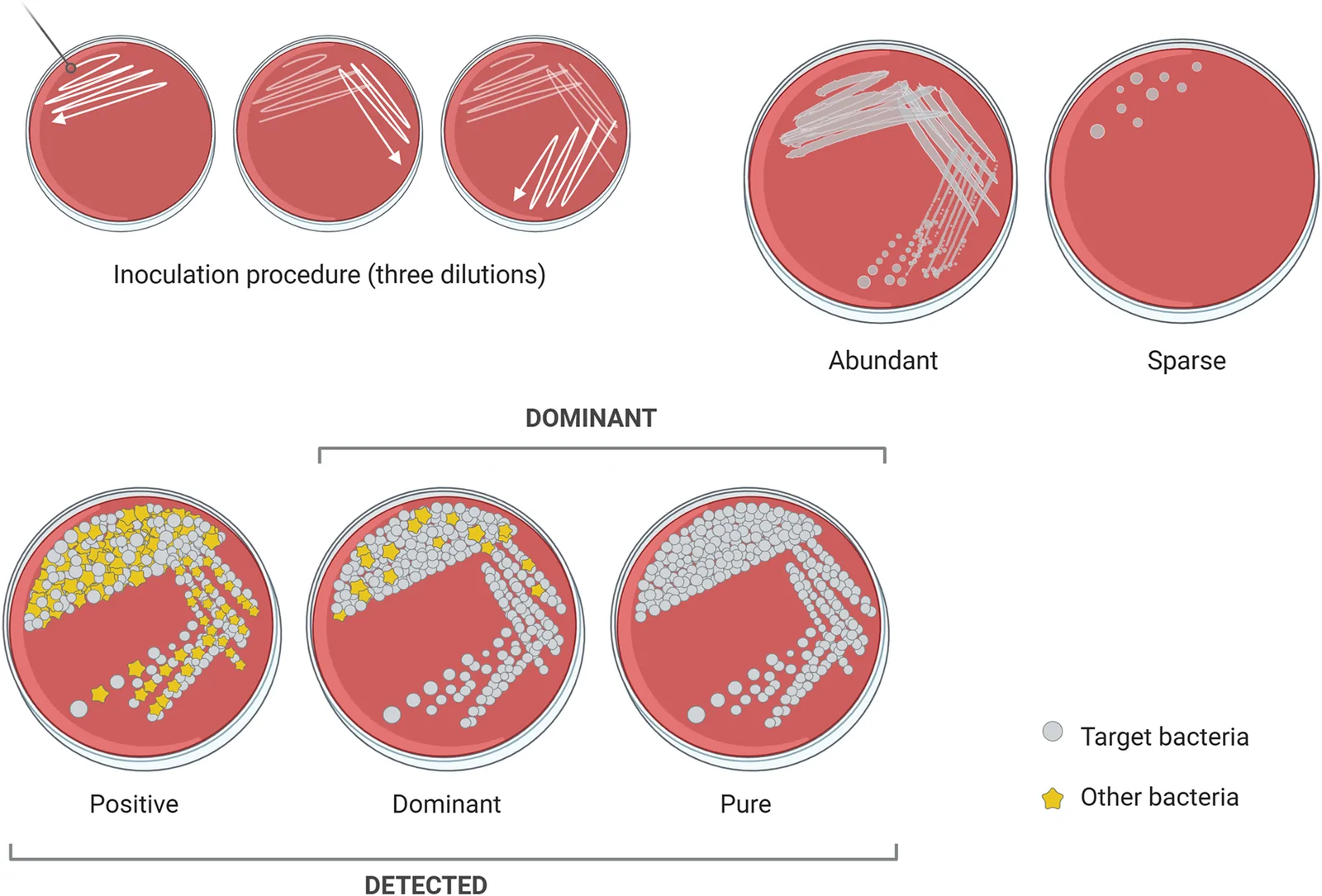
Schematic drawing of the bacterial inoculation procedure and semi-quantitative culture result definitions. The bottom three cultures illustrate how we described the composition of each culture, while the top-right cultures illustrate how we described the abundance of growth of those cultures. Culture results were merged into larger categories for analyses (shown in capital letters) and included the following: “Detected” = all cultures where target species were present (positive + dominant + pure); “dominant” = dominant and pure cultures, and cultures with ≥ 2 *Pasteurellaceae* spp. exclusively with equal growth (not included in drawing); and “dominant and abundant” = cultures with abundant growth within the “dominant” category. Figure created with BioRender.com

Figure 2 provides an overview of the descriptive and statistical analyses for *P. multocida*,* M. haemolytica*, and *H. somni*. A schematic drawing illustrating the main culture result definitions is provided in Fig. 3. We defined culture results as follows: negative (0 colonies of target species); positive (≥ 1 colony of target species); dominant (≥ 2 species where ≥ 1 species grow more abundantly than others); and pure (only 1 species). Co-occurring bacteria were defined as ≥ 2 *Pasteurellaceae* spp. detected (≥ 1 colony of each species). Bacterial growth was considered sparse in NS and NPS cultures if growth was present in ≤ half of the first dilution, and abundant if present in > half of the first dilution. In BAL cultures, sparse growth was < 10 colonies, and abundant was ≥ 10. Contamination of BAL samples from the upper airways was defined as < 10 colonies with ≥ 3 bacterial species where no species dominated, or any growth where contaminants (environmental bacteria or upper airway flora not associated with respiratory disease) were dominant. Enriched BAL cultures were described as a separate category from non-enriched BAL cultures (BAL).

All variables were analyzed as dichotomous categories. Culture results were categorized into the following gradients, from broad to narrow: (1) detected, (2) dominant, and (3) dominant and abundant. At the detection level, a sampling site was considered positive if the target species was present, regardless of dominance; an animal was positive if ≥ 1 sampling site was positive; and a herd was positive if ≥ 1 animal within the herd was positive. Dominant cultures were defined as dominant cultures, pure cultures and cultures composed ≥ 2 *Pasteurellaceae* spp. exclusively with equal growth. For the category dominant, abundant cultures, only dominant cultures with abundant growth were included. For viruses, a herd was positive if ≥ 1 animal within the herd tested positive. For AM susceptibility analysis, the categories susceptible, susceptible at increased exposure, and resistant were analyzed separately. To minimize bias, we merged the culture and AM susceptibility data with the health data after the culture results and clinical scoring were complete, ensuring blinding.

#### Statistical analyses

Descriptive and statistical analyses (Fig. 2) were performed using Stata/MP 15.1 (Stata Corp, College Station, TX). Pearson’s chi-squared test and Fisher’s exact test examined the univariate relationship between the outcome variable (i.e. health status) and predictor variables (e.g. detected bacteria). Culture results of *P* ≤ 0.20 were tested separately using random effects logistic regression models (*xtlogit*), adjusting for herd as a random effect. Additional predictor variables were age, as both a continuous and categorical variable (≤ 60 days and > 60 days), with the cut-off corresponding to the typical weaning age; breed (Norwegian Red and other breeds); and gender (male and female). Variables with *P* ≤ 0.20 or with known clinical importance, were included in the extended multivariable model. For the final model, we used the backward elimination method until all variables left in the model had *P* < 0.05.

Evaluation of upper airway sampling sites (NS and NPS) was conducted using BAL as the reference test. Agreement between upper airway sites and BAL for detection of bacteria was assessed by the percent positive agreement (PPA), Cohen’s kappa, and McNemar’s exact test. PPA between two sites was calculated as the number of calves positive by both sites divided by the average number of calves positive by either site, multiplied by 100 [32]. Confidence intervals (CI) for proportions were calculated using the Wald (normal approximation) method. Agreement based on the kappa statistic was interpreted according to the Landis and Koch scale: < 0.00 = poor; 0.00-0.20 = slight; 0.21–0.40 = fair; 0.41–0.60 = moderate; 0.61–0.80 = substantial; and 0.81-1.00 = almost perfect [39]. McNemar’s exact test was used to compare the marginal proportions of positive results between two sites [32]. Positive and negative predictive values (PPV and NPV) for NS and NPS relative to BAL were calculated as described [40]. PPV was defined as the proportion of true positives among all positive index test results, and NPV as the proportion of true negatives among all negative index test results. Estimates were calculated separately for each bacterial amount category.

#### Sample size calculation

Sample size calculations were based on the number of individuals needed to detect a difference between positive proportions of two groups with 80% power at a 5% significance level. *P. multocida* was assumed to be the most common pathogen, based on a previous pilot study [41]. To detect a 25-percentage point difference in positive proportions of a given culture result between healthy and diseased calves, assuming a 60% prevalence of *P. multocida* in the upper airway sites of diseased calves, a sample size of 62 calves per group was needed. In the lower airway site, a sample size of 76 calves per group was required to detect a 15-percentage point difference in positive proportions of dominant, abundant growth of *P. multocida*, assuming a 20% prevalence in diseased calves. Finally, we estimated a need for 117 calves with paired sampling sites for McNemar’s test to detect a 15-percentage point difference in positive proportions of *P. multocida*-positive cultures, assuming discordant results in 25% of cultures.

## Results

### Occurrence of BRD pathogens

#### *Pasteurellaceae* species isolated

From the 131 calves, we cultured 131 nasal swabs (NS), 131 nasopharyngeal swabs (NPS), and 113 non-endoscopic bronchoalveolar lavage samples (BAL). Eighteen calves were missing BAL samples: 10 calves in herd A (*n* = 10) due to limited sampling time, and 8 calves from other herds, due to unsuccessful attempts at accessing the lower airways. Fluid return was recorded for 93/113 BAL samples, showing a mean return of 33% of the instilled fluid (SD ± 13%; median 30% [range: 7–63%]). No clear differences in fluid return were observed between different culture results categories, regardless of volume instilled during BAL (Additional file 4). Sixty-two enriched BAL samples were cultured following bacteria negative cultures from the corresponding non-enriched BAL. Culture results at the herd, calf, and sampling site levels are summarized in Table 2.

**Table 2:** Occurrence of *Pasteurellaceae* species in 131 calves from nine dairy herds with enzootic pneumonia

Culture result	Percentages (*n*) of herds and calves with each culture result					
**Species**,** detected**	**Herd**^a^ (*n* = 9)	**Calf**^a^ (*n* = 131)	**NS** (*n* = 131)	**NPS** (*n* = 131)	**BAL** (*n* = 113)	**Enriched BAL**^b^ (*n* = 62)
*P. multocida*	89% (8)	60% (78)	47% (62)	45% (59)	27% (30)	29% (18)
*M. haemolytica*	100% (9)	55% (72)	46% (60)	37% (48)	8% (9)	10% (6)
*H. somni*	22% (2)	3% (4)	2% (2)	2% (2)	1% (1)	0% (0)
No *Pasteurellaceae* spp.	0% (0)	17% (22)	26% (34)	31% (41)	67% (76)	63% (39)
Combinations, detected^c,d^						
* P. multocida* + *M. haemolytica*	67% (6)	31% (41)	20% (26)	13% (17)	2% (2)	2% (1)
* P. multocida* + *H. somni*	0% (0)	3% (4)	1% (1)	2% (2)	1% (1)	0% (0)
* P. multocida* + *M. haemolytica* + *H. somni*	22% (2)	0% (0)	0% (0)	0% (0)	0% (0)	0% (0)
No combination of *Pasteurellaceae* spp.	11% (1)	66% (86)	79% (104)	85% (112)	97% (110)	98% (61)

*Abbreviations*: *NS*  Nasal swab, *NPS*  Nasopharyngeal swab, *BAL*  Bronchoalveolar lavage

^a﻿^Herds were classified as positive if ≥ 1 calf in the herd was positive; calves were classified as positive if ≥ 1 sampling site (NS, NPS, or BAL, including enriched BAL) was positive

^b﻿^Enriched BAL = BAL fluid enriched with BHI broth following bacteria negative culture from the corresponding non-enriched BAL fluid, providing additional isolates from the lower airways

^c﻿^For species combinations: Herds were classified as positive if all species in the combination were detected within the herd; calves were classified as positive if the combination occurred within the same calf (not necessarily from the same sampling site). Herds, calves, and sampling sites were assigned to only one combination category (e.g. a herd positive for a three-species combination was not also included in a two-species category)

^d﻿^No combinations of *M. haemolytica* or *H. somni* were detected

**Fig. 4:**
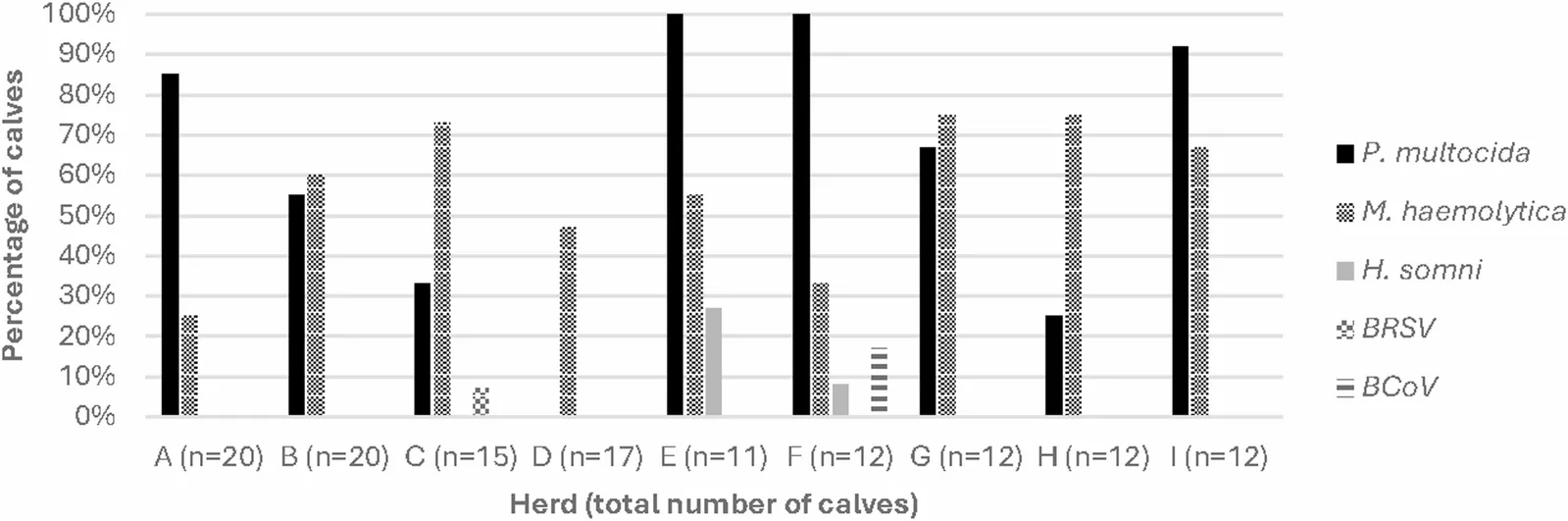
Occurrence of BRD-associated pathogens in calves per herd. The figure shows the percentage of calves per herd with BRD-associated pathogens detected in nasal swabs, nasopharyngeal swabs, or bronchoalveolar lavage samples among 131 calves in nine dairy herds with enzootic pneumonia

Within each herd, *P. multocida* and *M. haemolytica* were the most frequently isolated species (Fig. 4). At the calf level, *Pasteurellaceae* spp. were isolated in 83% (109/131) of calves. *P. multocida* was the most frequently isolated species overall, followed by *M. haemolytica*, while *H. somni* was rarely detected (Table 2). We identified ≥ 2 different *Pasteurellaceae* spp. in 34% (45/131) of calves. At the sampling site level, calves were *Pasteurellaceae* spp. positive in 74% (97/131) of NS, 69% (90/131) of NPS, and 33% (37/113) of BAL samples. Isolation rates for each species decreased from the upper (NS and NPS) to the lower airway sites (BAL). However, the decline was less pronounced for *P. multocida*, which had similar isolation rates to *M. haemolytica* in NS, but accounted for 81% (30/37) of *Pasteurellaceae* spp. positive BAL samples, compared to 24% (9/37) for *M. haemolytica*. *H. somni* was rarely detected in any of the sampling sites (≤ 2% positive per site), but was represented in each. The frequencies of co-occurring *Pasteurellaceae* spp., mainly *P. multocida* and *M. haemolytica*, also decreased from upper to lower airway sites, appearing in 21% (27/131) of NS, 15% (19/131) of NPS, and 3% (3/113) of BAL samples. Combinations of *M. haemolytica* and *H. somni*, or of all three *Pasteurellaceae* spp. described, were not detected at the sampling site level in any of the herds.

#### Other BRD-associated pathogens isolated

BRD-associated pathogens other than the three main *Pasteurellaceae* spp. were seldom predominant in a herd. The exception was herd D where we identified *T. pyogenes* in 65% of calves (11/17), including 47% (8/17) of NS, 18% (3/17) of NPS, 18% (3/17) of BAL (two as single-species), and 7% (1/14) of enriched BAL. In the same herd, the fungus *Lichtheimia corymbifera* was present in 76% (13/17) of NS, 12% (2/17) of NPS, and none of the BAL samples. *B. trehalosi* was identified only sporadically (one NS in herd C and one enriched BAL in herd D).

#### Serological results for *Mycoplasmopsis bovis*

We analyzed serum samples from 130 of the 131 calves (one sample was missing). All calves were negative for antibodies against *M. bovis*, with no doubtful samples that needed to be re-tested.

#### qPCR results for BRD viruses

RNA extracted from NS of the 131 calves underwent qPCR analysis. One calf from herd C tested positive for BRSV and two calves from herd F tested positive for BCoV (Fig. 4). Herd C and F were therefore regarded as BRD virus positive herds. All calves tested negative for PIV3, BVDV, and BHV1.

### Antimicrobial resistance pattern (AMR) of *Pasteurellaceae* species

#### Antimicrobial use in study herds

All farms frequently used procaine benzylpenicillin to treat BRD, five of which used it exclusively (farm A, B, E, H, I). In addition, florfenicol was frequently used to treat BRD in three farms (D, F, G), oxytetracycline in one farm (C), and gamithromycin in one farm (G).

#### Phenotypic antimicrobial resistance of *Pasteurellaceae *species

From the 131 calves, we assessed the phenotypic AMR pattern in 169 *P. multocida*, 123 *M. haemolytica*, and five *H. somni* isolates. All *Pasteurellaceae* spp. isolates were susceptible to amoxicillin-clavulanic acid, trimethoprim sulfamethoxazole, tetracycline, enrofloxacin, and florfenicol. Additionally, all *P. multocida* and *H. somni* isolates were susceptible to penicillin, while 4% (5/123) of *M. haemolytica* isolates were resistant, originating from both the upper and lower sampling sites in two calves (3% of *M. haemolytica*-positive calves), both from herd G. For streptomycin, 6% (10/169) of *P. multocida* isolates were susceptible, 1% (2/169) were susceptible at increased exposure, and 93% (157/169) were resistant, while for *M. haemolytica*, 72% (89/123) were susceptible, 6% (7/123) were susceptible at increased exposure, and 22% (27/123) were resistant. All five *H. somni* isolates were susceptible to streptomycin. Streptomycin-resistant *P. multocida* were found among 92% (72/78) of *P. multocida*-positive calves, including all eight herds where *P. multocida* was prevalent, while streptomycin-resistant *M. haemolytica* were detected in 26% (19/72) of *M. haemolytica*-positive calves, from seven out of nine positive herds.

The supplementary assessment comparing AMR patterns of isolates within the same sample and animal, including a total of 53 isolates from nine calves across six herds, found no variability in phenotypic AMR pattern among isolates (Additional file 5).

### Culture result interpretation

#### Detection in healthy vs. diseased calves

An overall clinical score classified 72 calves as healthy and 59 calves as diseased (Additional file 6). Among healthy and diseased calves, *P. multocida* was present in 56% (40/72) and 64% (38/59), *M. haemolytica* in 60% (43/72) and 49% (29/59), and *H. somni* in 1% (1/72) and 5% (3/59), respectively. Culture results from all sampling sites are presented in Table 3.

**Table 3:** Culture results of *Pasteurellaceae* species from sampling sites of healthy and diseased calves

Culture result		Nasal swabs % (*n*)			Nasopharyngeal swabs % (*n*)			Bronchoalveolar lavage % (*n*)		
**Species**	**Gradient** ^a^	**Total** (*n* = 131)	**Healthy** (*n* = 72)	**Diseased** (*n* = 59)	**Total** (*n* = 131)	**Healthy** (*n* = 72)	Diseased (*n* = 59)	**Total** (*n* = 113)	**Healthy** (*n* = 66)	**Diseased** (*n* = 47)
*P. multocida*	Detected	47% (62)	42% (30)	54% (32) *	45% (59)	38% (27)	54% (32) **	27% (30)	24% (16)	30% (14)
	Dominant	37% (49)	33% (24)	42% (25)	39% (51)	33% (24)	46% (27) *	22% (25)	20% (13)	26% (12)
	*- Pure* ^d^	*5% (6)*	*6% (4)*	*3% (2)*	*19% (25)*	*13% (9)*	*27% (16)*	*19% (22)*	*17% (11)*	*23% (11)*
	Dominant + abundant	36% (47)	31% (22)	42% (25) *	16% (21)	13% (9)	20% (12)	13% (15)^e^	8% (5)	21% (10) ***
*M. haemolytica*	Detected	46% (60)	49% (35)	42% (25)	37% (48)	39% (28)	34% (20)	8% (9)	9% (6)	6% (3)
	Dominant	21% (27)	26% (19) **	14% (8)	23% (30)	24% (17)	22% (13)	3% (3)	3% (2)	2% (1)
	*- Pure* ^d^	*0% (0)*	*0% (0)*	*0% (0)*	*3% (4)*	*6% (4)*	*0% (0)*	*1% (1)*	*2% (1)*	*0% (0)*
	Dominant + abundant	18% (23)	21% (15)	14% (8)	7% (9)	6% (4)	8% (5)	1% (1)	0% (0)	2% (1)
*H. somni* ^b^	Detected	2% (2)	0% (0)	3% (2)	2% (2)	0% (0)	3% (2)	1% (1)	2% (1)	0% (0)
*P. multocida +* *M. haemolytica* ^c^	Detected	20% (26)	18% (13)	22% (13)	13% (17)	13% (9)	14% (8)	2% (2)	3% (2)	0% (0)
No *Pasteurellaceae*	Detected	26% (34)	28% (20)	24% (14)	31% (41)	36% (26)	25% (15)	67% (76)	70% (46)	64% (30)

Culture results from healthy and diseased calves from nine dairy herds with enzootic pneumonia and their association with health status, as assessed by Pearson’s chi-squared (≥ 5 frequencies per group) or Fisher’s exact test (< 5 frequencies in one or both groups)

*Abbreviations*: *NS*  Nasal swab, *NPS*  Nasopharyngeal swab, *BAL*  Bronchoalveolar lavage

*P*-values are indicated as follows: no annotation = *P* > 0.20; * = *P* ≤ 0.20; ** = *P* ≤ 0.10; *** = *P* < 0.05

^a﻿^Detected = all positive cultures; dominant = all dominant and pure cultures; dominant + abundant = dominant with growth in > half of 1st dilution of culture (NS, NPS) or ≥ 10 colonies (BAL)

^b﻿^All *H. somni*-positive cultures were in combination with *P. multocida*, except in one NS, which was pure culture

^c﻿^Out of the co-occurring *P. multocida* and *M. haemolytica*, *P. multocida* was dominant in 77% (20/26), 88% (15/17), and 100% (2/2) of NS, NPS, and BAL, respectively, while *M. haemolytica* was dominant in 38% (10/26), 53% (9/17), and 50% (1/2) of NS, NPS, and BAL

^d﻿^Pure cultures were included in the “dominant” category for statistical analysis

^e﻿^Out of BAL cultures with dominant, abundant growth of *P. multocida*, 87% (13/15) were pure cultures

At detection level, Pearson’s chi-squared or Fisher’s exact test revealed no statistically significant associations between health status and the presence of any of the *Pasteurellaceae* spp., alone or in combination. However, we observed some trends. Isolation rates of *P. multocida* were consistently higher in diseased calves compared to healthy ones in cultures from all three sites, while for *M. haemolytica*, isolation rates were similar between the two groups. Moreover, in healthy calves, the occurrence of *P. multocida* and *M. haemolytica* was similar in NS and NPS cultures, while *P. multocida* was the predominant species in BAL. In diseased calves, *P. multocida* was the most frequently isolated species in all three sites.

In herds with predominantly healthy calves (herds B, C, and H), the median percentage of *P. multocida*-positive calves was 33% (range: 25–55%), which was lower than the overall median of 76% (range: 25–100%) across all positive herds (Additional file 7). The median percentage of calves with *P. multocida*-positive BAL cultures was 15% (range: 8–18%) in these herds, compared to 22% (range: 8–92%) across all positive herds.

#### Dominant cultures across species, sampling sites, and health status

As shown in Table 3, P. *multocida*-positive cultures were mostly dominant, making up 79% (49/62), 86% (51/59), and 83% (25/30) of positive cultures from NS, NPS, and BAL, respectively. In comparison, *M. haemolytica* was dominant in 45% (27/60), 63% (30/48), and 33% (3/9) of positive NS, NPS, and BAL cultures, respectively. Nearly all pure cultures were made up by *P. multocida.* Pure cultures comprised 10% (6/62), 42% (25/59), and 73% (22/30) of *P. multocida-*positive cultures from NS, NPS, and BAL, respectively.

A significantly higher proportion of diseased calves compared to healthy ones had dominant, abundant growth of *P. multocida* in BAL cultures (Chi^2^, *P* < 0.05). No other associations were found between health status and culture results in any of the three sites.

#### Associations between health status and predictor variables by logistic regression

Univariate models identified two variables associated with respiratory disease: dominant, abundant cultures of *P. multocida* from BAL and the calf being male (*P* < 0.05*)*. An overview of all tested variables is provided in Additional file 8. These two variables remained statistically significant in the multivariable model for respiratory disease (Table 4). Calves with dominant, abundant cultures of *P. multocida* from BAL had approximately 7-times higher odds of clinical disease compared to those with negative BAL cultures, and male calves had 3-times higher odds of disease compared to female calves.

**Table 4:** Multivariable model describing associations between health status and predictor variables in 113 dairy calves

Predictor variables^a^	Level (*n*)	β^b^	OR^b^	SE^b^	95% CI^b^	*P* ^b^
*P. multocida* dominant, abundant culture, BAL	Negative (98)	-	1.0	-	-	-
	Positive (15)	1.87	6.46	4.89	1.46–28.51	0.01
Gender	Female (59)	-	1.0	-	-	-
	Male (54)	1.34	3.38	1.74	1.57–9.33	< 0.01
Constant		-1.29	0.28	0.12	0.12–0.63	< 0.01

Multivariable model describing associations between health status (0 = healthy, baseline; 1 = diseased) and predictor variables in 113 calves (66 healthy and 47 diseased) from nine dairy herds with enzootic pneumonia. Herd was included as a random effect

*Abbreviation*: *BAL*  Bronchoalveolar lavage

^a^Baselines of predictor variables (categorical): *P. multocida* dominant, abundant culture in BAL = negative; Gender = female

^b^Model output: β = beta coefficient; OR = odds ratio; SE = standard error; CI = confidence interval; *P* = p-value; N = sample size

### Evaluation of upper relative to lower airway sampling sites

#### Agreement in bacterial detection between NS and NPS cultures and BAL

Due to the limited number of positive BAL cultures, analyses were done only for *P. multocida* and performed on all calves combined (*n* = 113), independent of health status.

In general, *P. multocida* was detected more frequently in NS and NPS than in BAL, with a high proportion of positive cultures from NS and NPS paired with negative BAL cultures (Table 5). Among the 30 calves with a *P. multocida*-positive BAL culture, 73% (22/30) were positive in all three sites (NS, NPS, and BAL), 10% (3/30) were positive in BAL and either NS or NPS, and 17% (5/30) were positive in BAL only. As shown in Table 5, culture results from NS and NPS were very similar and showed fair agreement with BAL and a significant McNemar’s test.

**Table 5:** Agreement between cultures from upper airway sites and BAL for detecting *P. multocida*

Upper airway site	Calves (*n*) per combination (BAL/upper airway site)				Percent positive agreement (95% CI)^a^	Kappa (95% CI)	Classification	*P* ^b^
	+/+	+/-	-/+	-/-				
NS (*n* = 113)	24	6	29	54	58 (47–68)	0.36 (0.28–0.45)	Fair	< 0.01
NPS (*n* = 113)	23	7	25	58	59 (48–70)	0.39 (0.30–0.48)	Fair	< 0.01

Results are based on 113 calves (66 healthy and 47 diseased) with paired sampling sites from nine dairy herds with enzootic pneumonia. Abbreviations: NS = nasal swab; NPS = nasopharyngeal swab; BAL = bronchoalveolar lavage

^a^The 95% confidence interval (CI) was calculated using the Wald (normal approximation) method

^b^P-value is based on McNemar’s exact test

#### Predictive values of semi-quantitative culture results in NS and NPS relative to BAL

Positive (PPV) and negative predictive values (NPV) of NS and NPS cultures relative to BAL are presented in Table 6. At the detection level, NS and NPS cultures showed similar predictive values: for predicting detection in BAL, PPV was at most 48%, while NPV was at least 89%; and for predicting dominant and abundant growth in BAL, PPV was at most 31%, while NPV was 100%. Across semi-quantitative categories in the upper airway sites, PPV and NPV in NS showed minimal variation. In NPS, however, both PPV and NPV decreased with increasingly narrow categories, regardless of the BAL reference category.

**Table 6:** Predictive values of *P. multocida* culture results in upper airway sites relative to BAL

			Culture result for *P*. multocida in BAL/upper airway site																	
			Detection in BAL						Dominant in BAL						Dominant, abundant in BAL					
Population	Upper airway site	Culture result for *P. multocida*	+/+	+/-	-/+	-/-	PPV %	NPV %	+/+	+/-	-/+	-/-	PPV %	NPV %	+/+	+/-	-/+	-/-	PPV %	NPV %
All calves (*n* = 113)	NS	Detected	24	6	29	54	45	90	23	2	30	58	43	97	15	0	38	60	28	100
		Dominant	22	8	23	60	49	88	22	3	23	65	49	96	15	0	30	68	33	100
		*- Pure* ^a^	*2*	*28*	*4*	*79*	***-***	***-***	*2*	*23*	*4*	*84*	*-*	*-*	*0*	*15*	*6*	*92*	*-*	*-*
		Dominant+ abundant	21	9	22	61	49	87	21	4	22	66	49	94	15	0	28	70	35	100
	NPS	Detected	23	7	25	58	48	89	22	3	26	62	46	95	15	0	33	65	31	100
		Dominant	16	14	25	58	39	81	16	9	25	63	39	88	10	5	31	67	24	93
		*- Pure* ^a^	*9*	*21*	*13*	*70*	*-*	*-*	*9*	*16*	*13*	*75*	*-*	*-*	*5*	*10*	*17*	*81*	*-*	*-*
		Dominant+ abundant	4	26	9	74	31	74	4	21	9	79	31	79	1	14	12	86	8	86

Cross-tabulation of *P. multocida* culture results in upper airway sites relative to BAL (BAL/upper airway site) and the corresponding predictive values, based on 113 calves (66 healthy and 47 diseased) with paired sampling sites from nine dairy herds with enzootic pneumonia. No statistical analyses were done for healthy and diseased calves separately due to insufficient sample size at the group level

Abbreviations: NS = nasal swab; NPS = nasopharyngeal swab; BAL = bronchoalveolar lavage; PPV = positive predictive value; NPV = negative predictive value

^a﻿^Pure cultures were analyzed as part of the “dominant” cultures category

The cross-tabulated culture results for upper and lower airway sites in healthy and diseased calves separately are presented in Additional file 9.

### Impact of isolates from enriched BAL

When the additional 24 isolates from enriched BAL samples were combined with those from non-enriched BAL, *Pasteurellaceae* spp. were present in BAL from 53% (60/113) of all calves, including *P. multocida* in 42% (48/113) of calves and *M. haemolytica* in 13% (15/113). At the health group level, *P. multocida* was detected in 38% (25/66) of healthy and 49% (23/47) of diseased calves, and *M. haemolytica* in 15% (10/66) of healthy and 11% (5/47) of diseased calves. No statistically significant differences were observed between healthy and diseased calves; thus, conclusions were the same with and without the inclusion of enriched BAL isolates.

### Impact of upper airway contamination

Among 37 BAL cultures positive for *Pasteurellaceae* spp., 11% (4/37), from which two *P. multocida* and two *M. haemolytica* isolates originated, contained potential upper airway contamination, based on our definition mentioned previously. Excluding these samples led to negligible changes. Out of 76 BAL cultures negative for *Pasteurellaceae* spp., 3% (2/76) were positive for *T. pyogenes*; 87% (66/76) had no bacterial growth or ≤ three individual colonies of contaminants; 8% (6/76) had ≥ four individual colonies of contaminants; and 3% (2/76) had non-interpretable polymicrobial growth. From enriched BAL, 17% (4/23) of positive cultures, also including two *P. multocida* and two *M. haemolytica* isolates, contained dominant growth of contaminants.

## Discussion

### Main aims

In this study, we aimed to assess the occurrence and AMR pattern of BRD-associated bacteria in calves from Norwegian dairy herds with enzootic pneumonia, examine the criteria by which *Pasteurellaceae* spp. are associated with disease, and determine whether cultures from NS and NPS are reliable proxies for lower airway cultures. This is the first systematic study of BRD-associated bacteria in Norway, a country where *M. bovis* has not been detected and where procaine benzylpenicillin is the primary treatment for BRD.

### Occurrence of BRD-associated pathogens

The relative occurrence of *Pasteurellaceae* spp. found in calves from Norwegian dairy herds with enzootic pneumonia, is consistent with several Scandinavian [34, 35, 42], other European [43–45], and North American [32, 46] studies involving dairy calves, including multiple studies reporting *P. multocida* as the most common pathogen in the lower airway sites [34, 35, 42, 43, 45]. These findings support the established understanding of *P. multocida* as a key pathogen in enzootic pneumonia of young calves [13]. The higher prevalence of *P. multocida* compared with *M. haemolytica* in the lower airways, despite their frequent co-occurrence in the upper airways, might indicate differences in pathogenic potential under the conditions of this study. For example, *P. multocida* may possess virulence factors such as the hyaluronic acid capsule that facilitate lower airway colonization [47]. *M. haemolytica* has been suggested to exist in biofilms in the upper airways of healthy calves, which may disperse in response to host stress chemicals [47, 48]. Stress levels in these young dairy calves may have been too low to trigger dispersal and movement to the lower airways. It is also possible that *M. haemolytica* strains in this study lacked key virulence factors needed to modulate the host immune system and promote lower airway colonization [47]; however, this was not investigated in the present study. The low occurrence of *H. somni* could be partly attributed to the use of culture-based detection, as slow-growing bacteria like *H. somni* are easily overgrown. Compared to PCR, which can detect small amounts of DNA from even dead bacteria, culture is less sensitive but likely more clinically relevant, as it detects only live bacteria.

Although common in Norway [10], BRSV and BCoV were detected at low rates. This may be attributed to the type of herds studied and the random timing of sampling, which may not have captured the acute stage of the disease. Since the exact stage of disease at the time of sampling was unknown, viral occurrence may have been underestimated due to sampling before or after viral shedding [38].

### Antimicrobial resistance pattern of *Pasteurellaceae* species

Nearly all *Pasteurellaceae* spp. isolates in this study were susceptible to commonly used AMs in BRD treatment. The low occurrence of penicillin resistance among studied bacteria has similarly been reported in other studies [25, 29, 49], including Scandinavian ones, but differ from Swiss and Polish studies [24, 50], which report higher frequencies. These differences highlight the importance of regional variability in AM susceptibility, which must be accounted for when selecting AM for treatment. The low resistance levels found among *Pasteurellaceae* spp. to common BRD AMs in this study, may result from a generally restrictive AM use in Norwegian dairy herds [51] and the widespread use of procaine benzylpenicillin as the first-choice AM to common diseases [23, 51]. Additionally, the small herd sizes in Norway facilitate close monitoring of individual calves, making a “wait and see” approach more feasible, and daily injections of penicillin less problematic. A German study highlighted similar benefits and found a higher proportion of multi-resistant *P. multocida* and *M. haemolytica* in larger herds, possibly due to less infectious disease control than in small herds [25]. In case of florfenicol and tetracycline, no resistance was detected among *Pasteurellaceae* spp. in this study. Low levels of resistance to florfenicol have similarly been observed in other studies [24, 25, 29, 44, 52], although higher resistance levels have also been described [53]. In contrast, tetracycline resistance is more commonly reported [24, 50, 52]. Regarding the less commonly used AMs, the frequent resistance to streptomycin in our study reflects its known prevalence among *Pasteurellaceae* spp [54, 55], driven by the wide occurrence of bacterial strains carrying plasmids with streptomycin resistance genes, maintained by selection pressure. We included streptomycin in our panel due to its previously common use in Norwegian calves.

Overall, the fact that we found almost no in vitro resistance to the common BRD AMs among *Pasteurellaceae* spp., in problem herds with greater risk of AMR development, supports the current assumptions of the national AM therapy guidelines. Although only one isolate per sampling site was subjected to AM susceptibility testing in this study, our supplementary assessment comparing AMR patterns within and between sites in a subset of calves indicates little variation among isolates. Additionally, our results, with mostly susceptible isolates, were considered robust, as the disc diffusion method has shown 100% specificity if expected resistance is low [56].

### Factors associated with respiratory disease

While more diseased calves were *P. multocida*-positive across all sampling sites, we found no significant associations between clinical disease and the detection of any of the *Pasteurellaceae* spp. Our findings support the current understanding that *Pasteurellaceae* spp. may colonize both the upper and lower airways in healthy animals [26, 42, 57]. The only culture result associated with disease was the dominant, abundant growth of *P. multocida* in BAL cultures, suggesting that bacterial amount may be useful to differentiate between colonization and true infection in the lungs. A study in dogs has similarly indicated that a threshold of higher bacterial counts in BAL can help distinguish commensal from clinically relevant growth in the lower respiratory tract [58]. In calves, findings are inconsistent: a Belgian study found no associations between bacterial counts of *Pasteurellaceae* spp. and disease [26], while a Danish study found an association between disease and high counts of *H. somni* and *M. haemolytica* [29]. Both studies reported high variation in bacterial amount across healthy and diseased calves. This variability may partly be attributed to the cross-sectional study design of these studies and to fluctuations in bacterial amount over the course of disease. Bacterial numbers may be low during early stages of infection and increase as disease progresses [26, 59]. In addition, BRD is multifactorial, and health outcomes are influenced not only by bacterial amount, but also by pathogen virulence, environmental factors, and the host immune system. For instance, studies have shown that some animals can tolerate a high bacterial amount with minimal clinical signs or lesions [30]. Therefore, abundant bacterial growth should be interpreted alongside other diagnostic findings that support a diagnosis of pneumonia, such as clinical signs, cytology, or ultrasonographic findings.

Another factor associated with respiratory disease was the calf being male. This contrasts with a U.S. study that found female calves to be more at risk for BRD [60]. In that study, female calves with BRD also had greater odds of failure of passive transfer of immunity. It is unclear whether this was related to colostrum management factors. Failure of passive transfer of immunity may also apply to male calves, as their higher birth weight and increased dystocia risk [61, 62] may lead to reduced colostrum intake. Another factor potentially putting males at greater BRD risk is their lower economical value in dairy herds, possibly leading to less investment in their treatment and care [62]. While the reason for the higher BRD risk in male calves is unknown in this study, potential explanations highlight the importance of proper calf management during the first weeks of life to prevent disease, regardless of gender.

### Comparison of culture results from upper and lower airway sampling sites

Although the blind BAL technique provides a sample from the lungs and was used as reference test, the method has some potential limitations. First, nasopharyngeal passage causes risk of contamination. In our study, few samples had contamination beyond a negligible amount, and more than half of the positive BAL cultures were pure cultures. This aligns with a Belgian study, which reported much less contamination in BAL samples compared to unguarded NPS [31], and could indicate that contamination is not a major issue. Second, the impact of fluid volume on quantitative results is uncertain as dilution adjustments were not done. The small variation in fluid return across culture results might suggest that fluid volume had minimal effect on our results. Finally, the blind BAL technique usually samples a single, random lung lobe. Particularly in sedated calves, it may systematically sample the diaphragmatic lobes, which tend to be less affected [63]. Although it is unclear whether this affects isolation rates, it may have led to some false negative results.

In this study, agreement (PPA and kappa) and McNemar’s test indicate that cultures from upper airway sites were not comparable to those of BAL for identifying *P. multocida*. Low PPVs for both NS and NPS further demonstrate poor prediction of bacterial presence in the lungs. Using stricter bacterial amount categories (e.g. dominant vs. detected) did not improve PPV, as the number of true positives were reduced similarly or more than false positives. This was true regardless of amount category used in the BAL reference test. Overall, this suggests that positive BAL cultures, with or without a high bacterial amount, were not consistently reflected by a specific amount in the upper airway sites, resulting in poor prediction of both bacterial presence and abundance in the lungs. In contrast, high NPVs indicate that negative upper airway cultures accurately predicted bacterial absence. This was partly due to the low number of positive BAL cultures paired with negative upper airway cultures, but also due to the large number of samples negative at both sites.

PPV, NPV, and kappa values are influenced by the true prevalence of the detected variable [40]. A lower prevalence of positive BAL cultures would yield a lower PPV and higher NPV, and vice versa. Since this prevalence may depend on health status [33], the accuracy of cultures from upper airway sites will differ from one population to another. This may partly explain the variability among studies, where agreement between cultures from upper and lower airway sites for *P. multocida* detection ranges from moderate or less [31, 34–36] to substantial or better [32, 33]. In the current population, the limited accuracy of NS and NPS cultures in identifying bacterial presence in BAL suggests that, if only one animal is being sampled, it might be better to sample the lower airways. Alternatively, sampling multiple calves in the upper airways could be considered, as a study in feedlot calves found that NPS was comparable to BAL for identifying BRD pathogens at the group level [36]. In either case, culture results must be interpreted carefully, in context of other diagnostic findings and BRD herd prevalence.

Previous studies have indicated that material from NS has limited value compared to NPS due to sampling of the cutaneous mucosa [27] and because the nasopharyngeal microbiota contributes more to the lower airway microbiota [64]. However, in this study, guarded NPS did not look superior to NS for predicting the presence and abundance of *P. multocida* in BAL. A previous study in dairy calves with acute BRD also found that NS and NPS were similar in their ability to identify bacteria in the lungs [32]. Our findings support that the cheaper and less laborious NS may provide culture results comparable to guarded NPS, at least for *P. multocida* at the phenotypic level.

### Limitations

While the absence of several BRD pathogens removed some risks of bias, other limitations remain that could influence the validity of our findings. For internal validity, which refers to the accuracy of results for the source population and the impact of systematic biases [40], the following should be considered: First, there was a potential risk of misclassifying healthy and diseased calves, as numerous calves had clinical scores near the clinical cut-off point, making results sensitive to the set threshold. This issue highlights a challenge in herds with enzootic pneumonia, where calves cannot necessarily be clearly distinguished as either healthy or diseased, due to varying disease stages and etiologies. Many calves may be chronic, subclinical, or at an intermediate stage. To minimize misclassification bias, we used the same examiner for most examinations. However, since calves were sampled and examined once, we cannot rule out that some healthy calves may have had subclinical pneumonia, potentially leading to an underestimation of bacterial impact. Other limitations, as previously mentioned, include the imperfect blind BAL technique as reference test. This test may have resulted in some false negatives due to systematic sampling of diaphragmatic lobes, biasing NS and NPS results toward lower correlation. Moreover, while calves treated with AMs within 14 days of sampling were excluded, earlier treatments might have reduced the bacterial amount in the lungs, which may have resulted in some diseased calves with lower bacterial amounts. A final limitation was the sample size. At the herd level, the sample size was relatively low. At the calf level, the resulting number of calves was similar to the estimated requirements. However, the difference in proportions of positive bacterial culture results between healthy and diseased calves (i.e. the effect size) was smaller than expected, particularly in the upper airway sampling sites. Therefore, the sample size may not have provided enough power to detect an association, or to confidently support its absence.

In terms of external validity, which refers to the generalizability of findings to the target population [40], we consider our results valid for Norwegian herds with enzootic pneumonia. However, the findings may not be directly generalizable to populations with different pathogens (e.g. *M. bovis*), other breeds, herd characteristics, or other environmental conditions.

## Conclusion

*P. multocida* was the predominant pathogen in calves from dairy herds with enzootic pneumonia, particularly in the lower airways. The only bacterial finding associated with clinical disease was the presence of dominant, abundant growth of *P. multocida* in the lungs. This may help distinguish true infection from colonization in clinically diseased calves. *M. haemolytica* and *H. somni* appeared to have less clinical impact under the conditions of this study. AMR appeared to not be a problem among studied *Pasteurellaceae* spp., with AMR-levels lower than those reported in most other countries. These findings emphasize that local knowledge of bacterial occurrence and AMR-levels may enable the use of mainly narrow-spectrum AMs in certain regions and contribute to preventing selection pressure. Bacterial presence and abundance in cultures from upper airway sites were unreliable predictors of bacterial presence and abundance in the lungs, suggesting that upper airway cultures must be interpreted carefully, in context of the herd BRD prevalence and with the aid of other diagnostic tools. This may ultimately reduce the misdiagnoses of bacterial pneumonia and unnecessary use of AMs.

## Supplementary Information


Supplementary Material 1.



Supplementary Material 2.



Supplementary Material 3.



Supplementary Material 4.



Supplementary Material 5.



Supplementary Material 6.



Supplementary Material 7.



Supplementary Material 8.



Supplementary Material 9.


## Data Availability

The datasets supporting the conclusions of this study are available from the corresponding author upon request.

## Abbreviations

Ab-ELISA: Antibody enzyme-linked immunosorbent assay
AMC: Amoxicillin-clavulanic acid
AM: Antimicrobial
AMR: Antimicrobial resistance
BAL: Bronchoalveolar lavage
BHI: Brain heart infusion broth
Enriched BAL: Bronchoalveolar lavage fluid enriched with brain heart infusion broth
BRD: Bovine Respiratory Disease
BCoV: Bovine coronavirus
BHV1: Bovine herpesvirus 1
BPI3: Bovine parainfluenzavirus Virus 3
BRSV: Bovine respiratory syncytial virus (new name: Bovine orthopneumovirus)
BVDV: Bovine viral diarrhea virus
CLSI: Clinical and Laboratory Standards Institute
Ct: Cycle threshold
ENR: Enrofloxacin
EUCAST: European Committee on Antimicrobial Susceptibility Testing
FFC: Florfenicol
MALDI-TOF MS: Matrix-assisted laser desorption/ionization- time-of-flight mass spectrometry
NMBU: Norwegian University of Life Sciences
NPS: Nasopharyngeal swab
NPV: Negative predictive value
NS: Nasal swab
NVI: Norwegian Veterinary Institute
PEN: Penicillin
PPV: Positive predictive value
qPCR: Quantitative polymerase chain reaction
STX: Trimethoprim + sulfamethoxazole
STREP: Streptomycin
TET: Tetracycline

## References

[1] Gorden PJ, Plummer P. Control, Management, and Prevention of Bovine Respiratory Disease in Dairy Calves and Cows. Veterinary Clin North America: Food Anim Pract. 2010;26(2):243–59. 10.1016/j.cvfa.2010.03.004 PMC7135383 20619182

[2] Griffin D. Economic Impact Associated with Respiratory Disease in Beef Cattle. Veterinary Clin North America: Food Anim Pract. 1997;13(3):367–77. 10.1016/s0749-0720(15)30302-9 9368983

[3] Pardon B, De Bleecker K, Hostens M, Callens J, Dewulf J, Deprez P. Longitudinal study on morbidity and mortality in white veal calves in Belgium. BMC Vet Res. 2012;8(1):26. 22414223 10.1186/1746-6148-8-26 PMC3366893

[4] Mikalsen V, Smistad M, Roalkvam T. Statistikksamling fra Ku- og Geitekontrollen 2024. Ås: Forskning & Fag, Norsk melkeråvare, TINE SA;; 2025.

[5] Buczinski S, Achard D, Timsit E. Effects of calfhood respiratory disease on health and performance of dairy cattle: A systematic review and meta-analysis. J Dairy Sci. 2021;104(7):8214–27. 33896639 10.3168/jds.2020-19941

[6] DeDonder KD, Apley MD. A literature review of antimicrobial resistance in Pathogens associated with bovine respiratory disease. Anim Health Res Reviews. 2015;16(2):125–34. 10.1017/S146625231500016X 26373635

[7] Guardabassi L, Apley M, Olsen JE, Toutain P-L, Weese S. Optimization of Antimicrobial Treatment to Minimize Resistance Selection. Antimicrobial Resistance in Bacteria from Livestock and Companion. Animals. 2018:637–73. 10.1128/microbiolspec.arba-0018-2017 PMC11633575 29932044

[8] Mosier D. Review of BRD pathogenesis: the old and the new. Anim Health Res Reviews. 2015;15(2):166–8. 10.1017/S1466252314000176 25351390

[9] Grissett GP, White BJ, Larson RL. Structured Literature Review of Responses of Cattle to Viral and Bacterial Pathogens Causing Bovine Respiratory Disease Complex. J Vet Intern Med. 2015;29(3):770–80. 25929158 10.1111/jvim.12597 PMC4895424

[10] Toftaker I, Sanchez J, Stokstad M, Nødtvedt A. Bovine respiratory syncytial virus and bovine coronavirus antibodies in bulk tank milk – risk factors and spatial analysis. Prev Vet Med. 2016;133:73–83. 27720029 10.1016/j.prevetmed.2016.09.003 PMC7114080

[11] Ånestad LM, Åkerstedt J, Dalaker J, Klevar S. The surveillance programme for bovine viral diarrhoea (BVD) in Norway 2024. Ås: Norwegian Veterinary Institute; 2025.

[12] Ånestad LM, Åkerstedt J, Dalaker J, Klevar S. The surveillance programme for infectious bovine rhinotracheitis (IBR) and infectious pustular vulvovaginitis (IPV) in Norway 2024. Ås: Norwegian Veterinary Institute; 2025.

[13] Griffin D. Bovine Pasteurellosis and Other Bacterial Infections of the Respiratory Tract. Veterinary Clin North America: Food Anim Pract. 2010;26(1):57–71. 10.1016/j.cvfa.2009.10.010 20117542

[14] Ånestad LM, Moldal T, Hopp P, Klevar S. The surveillance programme for *Mycoplasma bovis* in Norway 2021. Ås: Norwegian Veterinary Institute; 2022.

[15] Klem TB, Nilssen NR, Nordstoga AB, Kampen AH, Hopp P, Sølverød L, et al. Overvåkingsprogram 2019 - Smittemessige konsekvenser av økt grovfôrimport i 2018. Ås: Norwegian Veterinary Institute; 2020.

[16] Gulliksen SM, Jor E, Lie KI, Løken T, Åkerstedt J, Østerås O. Respiratory infections in Norwegian dairy calves. J Dairy Sci. 2009;92(10):5139–46. 19762832 10.3168/jds.2009-2224 PMC7126448

[17] Hurri E, Ohlson A, Lundberg Å, Aspán A, Pedersen K, Tråvén M. Herd-level prevalence of *Mycoplasma bovis* in Swedish dairy herds determined by antibody ELISA and PCR on bulk tank milk and herd characteristics associated with seropositivity. J Dairy Sci. 2022;105(9):7764–72. 35879164 10.3168/jds.2021-21390

[18] Vähänikkilä N, Pohjanvirta T, Haapala V, Simojoki H, Soveri T, Browning GF, et al. Characterisation of the course of Mycoplasma bovis infection in naturally infected dairy herds. Vet Microbiol. 2019;231:107–15. 30955796 10.1016/j.vetmic.2019.03.007

[19] Tardy F, Aspan A, Autio T, Ridley A, Tricot A, Colin A, et al. Mycoplasma bovis in Nordic European Countries: Emergence and Dominance of a New Clone. Pathogens. 2020;9(11):875. 33114269 10.3390/pathogens9110875 PMC7716209

[20] Gupta RS, Sawnani S, Adeolu M, Alnajar S, Oren A. Phylogenetic framework for the phylum Tenericutes based on genome sequence data: proposal for the creation of a new order Mycoplasmoidales ord. nov., containing two new families Mycoplasmoidaceae fam. nov. and Metamycoplasmataceae fam. nov. harbouring Eperythrozoon, Ureaplasma and five novel genera. Antonie Van Leeuwenhoek. 2018;111(9):1583–630. 29556819 10.1007/s10482-018-1047-3

[21] Andrés-Lasheras S, Jelinski M, Zaheer R, McAllister TA. Bovine Respiratory Disease: Conventional to Culture-Independent Approaches to Studying Antimicrobial Resistance in North America. Antibiotics. 2022;11(4):487. 35453238 10.3390/antibiotics11040487 PMC9025279

[22] Welling V, Lundeheim N, Bengtsson B. A Pilot Study in Sweden on Efficacy of Benzylpenicillin, Oxytetracycline, and Florfenicol in Treatment of Acute Undifferentiated Respiratory Disease in Calves. Antibiotics. 2020;9(11):736. 33114681 10.3390/antibiotics9110736 PMC7692533

[23] Terapianbefaling. Bruk av antibakterielle midler til produksjonsdyr: Statens legemiddelverk; 2023. Available from: https://www.dmp.no/veterinermedisin/terapianbefalinger-og-forskrivning-av-legemidler-til-dyr/bruk-av-antibakterielle-midler-til-matproduserende-dyr/terapianbefalinger-for-storfe. [cited 2026 Mar 4].

[24] Schönecker L, Schnyder P, Schüpbach-Regula G, Meylan M, Overesch G. Prevalence and antimicrobial resistance of opportunistic pathogens associated with bovine respiratory disease isolated from nasopharyngeal swabs of veal calves in Switzerland. Prev Vet Med. 2020;185:105182. 33152661 10.1016/j.prevetmed.2020.105182

[25] Melchner A, van de Berg S, Scuda N, Feuerstein A, Hanczaruk M, Schumacher M, et al. Antimicrobial Resistance in Isolates from Cattle with Bovine Respiratory Disease in Bavaria. Ger Antibiot. 2021;10(12):1538. 10.3390/antibiotics10121538 PMC8698709 34943750

[26] Van Leenen K, Van Driessche L, De Cremer L, Masmeijer C, Boyen F, Deprez P, et al. Comparison of bronchoalveolar lavage fluid bacteriology and cytology in calves classified based on combined clinical scoring and lung ultrasonography. Prev Vet Med. 2020;176:104901. 32014683 10.1016/j.prevetmed.2020.104901

[27] Pardon B, Buczinski S. Bovine Respiratory Disease Diagnosis: What Progress Has Been Made in Infectious Diagnosis? Veterinary Clinics. Food Anim Pract. 2020;36(2):425–44. 10.1016/j.cvfa.2020.03.005 PMC7244442 32451034

[28] Dabo SM, Taylor JD, Confer AW. Pasteurella multocida and bovine respiratory disease. Anim Health Res Reviews. 2007;8(2):129–50. 10.1017/S1466252307001399 18218157

[29] Kudirkiene E, Aagaard AK, Schmidt LMB, Pansri P, Krogh KM, Olsen JE. Occurrence of major and minor pathogens in calves diagnosed with bovine respiratory disease. Vet Microbiol. 2021;259:109135. 34090248 10.1016/j.vetmic.2021.109135

[30] Bassel LL, Tabatabaei S, Caswell JL. Host Tolerance to Infection with the Bacteria that Cause Bovine Respiratory Disease. Veterinary Clinics: Food Anim Pract. 2020;36(2):349–59. 10.1016/j.cvfa.2020.03.003 32451029

[31] Van Driessche L, Valgaeren BR, Gille L, Boyen F, Ducatelle R, Haesebrouck F, et al. A Deep Nasopharyngeal Swab Versus Nonendoscopic Bronchoalveolar Lavage for Isolation of Bacterial Pathogens from Preweaned Calves With Respiratory Disease. J Vet Intern Med. 2017;31(3):946–53. 28425146 10.1111/jvim.14668 PMC5435039

[32] Doyle D, Credille B, Lehenbauer TW, Berghaus R, Aly SS, Champagne J, et al. Agreement Among 4 Sampling Methods to Identify Respiratory Pathogens in Dairy Calves with Acute Bovine Respiratory Disease. J Vet Intern Med. 2017;31(3):954–9. 28295570 10.1111/jvim.14683 PMC5434980

[33] Capik SF, White BJ, Lubbers BV, Apley MD, DeDonder KD, Larson RL, et al. Comparison of the diagnostic performance of bacterial culture of nasopharyngeal swab and bronchoalveolar lavage fluid samples obtained from calves with bovine respiratory disease. Am J Vet Res. 2017;78(3):350–8. 28240958 10.2460/ajvr.78.3.350

[34] Myrenås M, Pringle M, Harbom B, Bengtsson B. Pasteurella multocida from deep nasal swabs and tracheobronchial lavage in bovine calves from Sweden. Acta Vet Scand. 2024;66(1):58. 39501282 10.1186/s13028-024-00781-7 PMC11539715

[35] Otten ND, Goecke NB, Michelsen AM, Nielsen LR, Capion N, Martin HL, et al. Comparing Occurrence of Bovine Respiratory Pathogens Detected by High-Throughput Real-Time PCR in Nasal Swabs and Non-Endoscopic Bronchoalveolar Lavage Samples from Dairy and Veal Calves. Pathogens. 2024;13(6):479. 38921777 10.3390/pathogens13060479 PMC11206406

[36] Allen JW, Viel L, Bateman KG, Rosendal S, Shewen PE, Physick-Sheard P. The microbial flora of the respiratory tract in feedlot calves: associations between nasopharyngeal and bronchoalveolar lavage cultures. Can J Vet Res. 1991;55(4):341–6. 1790489 PMC1263480

[37] Mikalsen V, Østerås O, Roalkvam T. Årsrapport fra Helsekortordningen 2020 - Statistikksamling fra Ku- og Geitekontrollen 2020. Ås: TINE Rådgiving/Helsestjenesten for storfe; 2021.

[38] Klem TB, Sjurseth SK, Sviland S, Gjerset B, Myrmel M, Stokstad M. Bovine respiratory syncytial virus in experimentally exposed and rechallenged calves; viral shedding related to clinical signs and the potential for transmission. BMC Vet Res. 2019;15(1):156. 31109324 10.1186/s12917-019-1911-z PMC6528318

[39] Landis JR, Koch GG. The Measurement of Observer Agreement for Categorical Data. Biometrics. 1977;33(1):159–74. 843571

[40] Dohoo I, Martin W, Stryhn H. 2nd ed. Charlottetown, PE, Canada: VER Inc; 2014.

[41] Kroknes MB, Langedal MS. Occurence, relevance and antibiotic resistance of Pasteurella multocida in the upper respiratory tract of calves. Oslo: Norwegian University of Life Sciences (NMBU); 2020.

[42] Angen Ø, Thomsen J, Larsen LE, Larsen J, Kokotovic B, Heegaard PMH, et al. Respiratory disease in calves: Microbiological investigations on trans-tracheally aspirated bronchoalveolar fluid and acute phase protein response. Vet Microbiol. 2009;137(1):165–71. 19186010 10.1016/j.vetmic.2008.12.024 PMC7117372

[43] Nikunen S, Härtel H, Orro T, Neuvonen E, Tanskanen R, Kivelä SL, et al. Association of bovine respiratory disease with clinical status and acute phase proteins in calves. Comp Immunol Microbiol Infect Dis. 2007;30(3):143–51. 17258318 10.1016/j.cimid.2006.11.004 PMC7132380

[44] Catry B, Haesebrouck F, Vliegher SD, Feyen B, Vanrobaeys M, Opsomer G, et al. Variability in Acquired Resistance of Pasteurella and Mannheimia Isolates from the Nasopharynx of Calves, with Particular Reference to Different Herd Types. Microb Drug Resist. 2005;11(4):387–94. 16359200 10.1089/mdr.2005.11.387

[45] Lachowicz-Wolak A, Klimowicz-Bodys MD, Płoneczka-Janeczko K, Bykowy M, Siedlecka M, Cinciała J, et al. The Prevalence, Coexistence, and Correlations between Seven Pathogens Detected by a PCR Method from South-Western Poland Dairy Cattle Suffering from Bovine Respiratory Disease. Microorganisms. 2022;10(8):1487. 35893545 10.3390/microorganisms10081487 PMC9332621

[46] Francoz D, Buczinski S, Bélanger AM, Forté G, Labrecque O, Tremblay D, et al. Respiratory Pathogens in Québec Dairy Calves and Their Relationship with Clinical Status, Lung Consolidation, and Average Daily Gain. J Vet Intern Med. 2015;29(1):381–7. 25619524 10.1111/jvim.12531 PMC4858077

[47] Snyder E, Credille B. *Mannheimia haemolytica* and *Pasteurella multocida* in Bovine Respiratory Disease: How Are They Changing in Response to Efforts to Control Them? Veterinary Clinics. Food Anim Pract. 2020;36(2):253–68. 10.1016/j.cvfa.2020.02.001 32327253

[48] Pillai DK, Cha E, Mosier D. Role of the stress-associated chemicals norepinephrine, epinephrine and substance P in dispersal of Mannheimia haemolytica from biofilms. Vet Microbiol. 2018;215:11–7. 29426400 10.1016/j.vetmic.2017.11.025

[49] FINRES-Vet 2023 - Finnish Veterinary Antimicrobial Resistance Monitoring and Consumption of Antimicrobial Agents. Seinäjoki, Finland: Finnish Food Authority; 2024.

[50] Lachowicz-Wolak A, Chmielina A, Przychodniak I, Karwańska M, Siedlecka M, Klimowicz-Bodys M, et al. Antimicrobial-Resistance and Virulence-Associated Genes of Pasteurella multocida and Mannheimia haemolytica Isolated from Polish Dairy Calves with Symptoms of Bovine Respiratory Disease. Microorganisms. 2025;13(3):491. 40142384 10.3390/microorganisms13030491 PMC11944906

[51] Rajala-Schultz P, Nødtvedt A, Halasa T, Persson Waller K. Prudent Use of Antibiotics in Dairy Cows: The Nordic Approach to Udder Health. Front Veterinary Sci. 2021;8.1–7. 10.3389/fvets.2021.623998 PMC7973009 33748209

[52] Timsit E, Hallewell J, Booker C, Tison N, Amat S, Alexander TW. Prevalence and antimicrobial susceptibility of Mannheimia haemolytica, Pasteurella multocida, and Histophilus somni isolated from the lower respiratory tract of healthy feedlot cattle and those diagnosed with bovine respiratory disease. Vet Microbiol. 2017;208:118–25. 28888626 10.1016/j.vetmic.2017.07.013

[53] Credille BC, Capik SF, Credille A, Crossley BM, Blanchard P, Woolums AR, et al. Agreement of antimicrobial susceptibility testing of Pasteurella multocida and Mannheimia haemolytica isolates from preweaned dairy calves with bovine respiratory disease. Am J Vet Res. 2023;84(11):ajvr23060140. 10.2460/ajvr.23.06.0140 37558231

[54] Michael Geovana B, Bossé Janine T, Schwarz S. Antimicrobial Resistance in Pasteurellaceae of Veterinary Origin. Microbiol Spectr. 2018;6(3). 10.1128/microbiolspec.arba-0022-2017. PMC11633590 29916344

[55] Schwarz S, Spies U, Schäfer F, Blobel H. Isolation and interspecies-transfer of a plasmid from Pasteurella multocida encoding for streptomycin resistance. Med Microbiol Immunol. 1989;178(2):121–5. 2733634 10.1007/BF00203308

[56] Catry B, Dewulf J, de Kruif A, Vanrobaeys M, Haesebrouck F, Decostere A. Accuracy of Susceptibility Testing of Pasteurella multocida and Mannheimia haemolytica. Microb Drug Resist. 2007;13(3):204–11. 17949308 10.1089/mdr.2007.721

[57] Griffin D, Chengappa MM, Kuszak J, McVey DS. Bacterial Pathogens of the Bovine Respiratory Disease Complex. Veterinary Clin North America: Food Anim Pract. 2010;26(2):381–94. 10.1016/j.cvfa.2010.04.004 20619191

[58] Peeters DE, McKiernan BC, Weisiger RM, Schaeffer DJ, Clercx C. Quantitative Bacterial Cultures and Cytological Examination of Bronchoalveolar Lavage Specimens in Dogs. J Vet Intern Med. 2000;14(5):534–41. 11012118 10.1892/0891-6640(2000)014<0534:qbcace>2.3.co;2

[59] Ackermann MR, Gallup JM, Zabner J, Evans RB, Brockus CW, Meyerholz DK, et al. Differential expression of sheep beta-defensin-1 and – 2 and interleukin 8 during acute Mannheimia haemolytica pneumonia. Microb Pathog. 2004;37(1):21–7. 15194156 10.1016/j.micpath.2004.04.003

[60] Deepak, Aly SS, Love WJ, Blanchard PC, Crossley B, Van Eenennaam AL, et al. Etiology and risk factors for bovine respiratory disease in pre-weaned calves on California dairies and calf ranches. Prev Vet Med. 2021;197:105506. 34740025 10.1016/j.prevetmed.2021.105506

[61] Linden TC, Bicalho RC, Nydam DV. Calf birth weight and its association with calf and cow survivability, disease incidence, reproductive performance, and milk production. J Dairy Sci. 2009;92(6):2580–8. 19447990 10.3168/jds.2008-1603

[62] Dachrodt L, Arndt H, Bartel A, Kellermann LM, Tautenhahn A, Volkmann M, et al. Prevalence of disorders in preweaned dairy calves from 731 dairies in Germany: A cross-sectional study. J Dairy Sci. 2021;104(8):9037–51. 33985777 10.3168/jds.2021-20283

[63] Van Driessche L, Valgaeren B, De Schutter P, Gille L, Boyen F, Ducatelle R, et al. editors. Effect of sedation on the intrapulmonary position of a bronchoalveolar lavage catheter in calves. 29th World Buiatrics Conference (WBC 2016); 2016. 10.1136/vr.103676 27114405

[64] Timsit E, McMullen C, Amat S, Alexander TW. Respiratory Bacterial Microbiota in Cattle: From Development to Modulation to Enhance Respiratory Health. Veterinary Clinics: Food Anim Pract. 2020;36(2):297–320. 10.1016/j.cvfa.2020.03.001 32451027

