# Supplementary material for: Cross-sectional study of calves from Norwegian dairy herds with enzootic pneumonia: pathogen occurrence, antimicrobial resistance, culture result interpretation, and sampling site agreement
Source: BMC Vet Res. 2026 May 18;22:503. doi: 10.1186/s12917-026-05557-x (PMC13523226; doi:10.1186/s12917-026-05557-x)
Supplement: Supplementary file 1 [file 12917_2026_5557_MOESM1_ESM.docx]

**Additional file 1**

**Table S1:** Key information of dairy herds and enrolled calves

| **Farm data** | | | | | | | |
| --- | --- | --- | --- | --- | --- | --- | --- |
| **Background** | | | | | **Calves sampled** | | |
| ***Farm ID*** | ***Region in Southern Norway*** | ***Herd size***  ***(cow-years)*** | ***No. of BRD diagnoses per year (no. of unique individuals)^1^***  *Year 1 Year 2* | | ***No. of calves sampled* (no. of pens)^2^** | ***Age (days)***  Median (range) | ***Weight (kg)^3^***  Median (range) |
| A | South-east | 62.6 | 11 (10) | 23 (20) | 20 (4) | 83 (11-293) | 111 (46-291) |
| B | South-east | 61.2 | 33 (25) | 47 (36) | 20 (3) | 76.5 (25-107) | 95 (63-119) |
| C | South-west | 109 | 56 (45) | 47 (42) | 15 (5) | 82 (30-116) | 107 (67-160) |
| D | North-west | 102.5 | 11 (10) | 31 (25) | 17 (3) | 50 (33-68) | 69 (50-95) |
| E | South-east | 44.1 | 21 (21) | 38 (29) | 11 (5) | 64 (11-85) | 81 (61-110) |
| F | North-east | 79 | 14 (11) | 24 (21) | 12 (2) | 66 (22-86) | 84 (63-92) |
| G | North-east | 122.1 | 65 (58) | 32 (26) | 12 (2) | 60 (19-142) | 80 (50-116) |
| H^4^ | South-east | 58.3 | 2 (1) | 5 (5) | 12 (2) | 54 (13-98) | 87 (51-119) |
| I | North-west | 85.5 | 12 (10) | 16 (15) | 12 (1) | 55.5 (30-82) | 77 (65-107) |
| All farms (A-I)^5^ |  | 79 (44.1-122.1) | 14 (2-65)  11 (1-58)^6^ | 31 (5-47)  25 (5-42)^6^ | 131 (27) | 64 (11-293) | 83 (46-291) |

Key data from nine dairy herds with enzootic pneumonia and 131 calves enrolled in the study.

^1^The number of diagnoses may include multiple registrations for the same calf; the number in parentheses represents the number of unique calves affected.

^2^All sampled pens were located in the same building for each herd. All pens were connected to each other through gridded fences, except in herd B where they were in three separate locations in the barn.

^3^Weight of calves was measured using a weight tape.

^4^Farm H was recruited last minute to replace a farm that cancelled. Although it did not meet the inclusion criteria of 10 calves diagnosed with BRD per year, it was included because calves were regularly diagnosed with BRD.

^5^Summary values are presented as median (range), except for number of calves and pens sampled, which are presented as totals.

^6^For BRD diagnoses, the first line indicates the median (range) number of diagnoses and the second line indicates the median (range) number of unique individuals.
