# Supplementary material for: Cross-sectional study of calves from Norwegian dairy herds with enzootic pneumonia: pathogen occurrence, antimicrobial resistance, culture result interpretation, and sampling site agreement
Source: BMC Vet Res. 2026 May 18;22:503. doi: 10.1186/s12917-026-05557-x (PMC13523226; doi:10.1186/s12917-026-05557-x)
Supplement: Supplementary file 2 [file 12917_2026_5557_MOESM2_ESM.docx]

**Additional file 2**

**Table S2:** Pre-visit farmer interview regarding general herd data, health history, and management.

|  | **Question** |
| --- | --- |
| **Participation** | - Would you like to participate in this project? - Are you a member of the Norwegian Dairy Herd Recording System (NDHRS) |
| **Production** | - Could you describe the herd and the type of production you have?   - ***Follow-up questions if needed:***     - Is this exclusively a dairy herd?     - How large is the herd? (cow years/yearly milk yield?)     - Do you have year-round or concentrated calving?     - Do you purchase live cattle?     - Do you have tie-stall or loose housing in the barn? How many operational buildings are used for the herd? - Can you describe the housing conditions for the different age groups in the herd, particularly for the calves? - Can you describe the feeding routines for the different age groups? |
| **Health** | - Can you describe the occurrence of BRD in the herd in general and over the last two years?   - ***Follow-up questions if needed:***     - Do you have the impression of a persisting BRD problem in the herd?     - How many calves are treated yearly? Which age groups are treated? - How has the occurrence of BRD changed? Is the problem increasing or decreasing? - Is BRD a seasonal problem in the herd? - To what extent do you notice mild cough in the calves throughout the year that does not warrant veterinary treatment? - Can you describe the occurrence of other health challenges in the herd, both in general and over the past two years? - Can you describe the antimicrobial use in the herd?   - ***Follow-up questions if needed:***     - Which antimicrobial drugs are used in the herd? - Do you vaccinate the calves? If so, which vaccination regimen is used? - Has anyone tried to solve the BRD issue in the herd? - Does the herd have regular visits from a veterinary practitioner or health advisor? |
| **Other** | - Is there any other relevant information you would like to add? |

**Table S3:** On-site observations during the herd visit.

|  | **Observations/measurements** |
| --- | --- |
| **Anamnesis** | Calf management routines |
|  | Impression of BRD problem (farmer’s and investigator’s observations) |
| **Housing** | Number of individuals per pen |
|  | Age range per pen |
|  | All-in/all-out system (yes or no) |
|  | Hygienic conditions |
|  | Flooring |
| **Feeding routines** | Milk feeding routines |
|  | Type and amount of roughage and concentrate |
| **Bedding material** | Type, amount, and quality |
| **Environmental measurements** | Temperature |
|  | Humidity |
|  | Air quality and the presence of drafts |
