# Supplementary material for: Cross-sectional study of calves from Norwegian dairy herds with enzootic pneumonia: pathogen occurrence, antimicrobial resistance, culture result interpretation, and sampling site agreement
Source: BMC Vet Res. 2026 May 18;22:503. doi: 10.1186/s12917-026-05557-x (PMC13523226; doi:10.1186/s12917-026-05557-x)
Supplement: Supplementary file 3 [file 12917_2026_5557_MOESM3_ESM.docx]

**Additional file 3**

**Table S4:** Antimicrobial panel with interpretative categories and inhibition zone diameter breakpoints for *Pasteurellaceae* spp.

|  |  | Inhibition zone diameters (mm) | | |  |
| --- | --- | --- | --- | --- | --- |
| **Antimicrobial agent** | **Disc content, µg** | **Susceptible** | **Susceptible, increased exposure** | **Resistant** | **Reference** |
| Benzylpenicillin | 10 | ≥ 17 | - | ≤ 16 | EUCAST, Clinical breakpoints (v14.0), 2024 |
| Amoxicillin-clavulanic acid | 30 | ≥ 15 | - | ≤ 14 | EUCAST, Clinical breakpoints (v14.0), 2024 |
| Trimethoprim + Sulfamethoxazole | 1.25/23.75 | ≥ 23 | - | ≤ 22 | EUCAST, Clinical breakpoints (v14.0), 2024 |
| Tetracycline | 30 | ≥ 24 | - | ≤ 23 | EUCAST, Clinical breakpoints (v14.0), 2024 |
| Enrofloxacin | 5 | ≥ 21 | 17-20 | ≤ 16 | CLSI, VET01S ED7, 2024 |
| Florfenicol | 30 | ≥ 19 | 15-18 | ≤ 14 | CLSI, VET01S ED7, 2024 |
| Streptomycin^1^ | 10 | ≥ 15 | 12-14 | ≤ 11 | CLSI, M100-ED34, 2024 |

Abbreviations: EUCAST = European Committee on Antimicrobial Susceptibility Testing; CLSI = Clinical and Laboratory Standards Institute.

^1^For streptomycin, EUCAST and CLSI do not define breakpoints for *Pasteurellaceae* spp. and we therefore applied those of *Enterobacteriaceae* spp. instead, as done in a previous study (1).

**References**

1.	Maynou G, Bach A, Terré M. Feeding of waste milk to Holstein calves affects antimicrobial resistance of Escherichia coli and Pasteurella multocida isolated from fecal and nasal swabs. Journal of Dairy Science. 2017;100(4):2682-94.
