# Supplementary material for: Cross-sectional study of calves from Norwegian dairy herds with enzootic pneumonia: pathogen occurrence, antimicrobial resistance, culture result interpretation, and sampling site agreement
Source: BMC Vet Res. 2026 May 18;22:503. doi: 10.1186/s12917-026-05557-x (PMC13523226; doi:10.1186/s12917-026-05557-x)
Supplement: Supplementary file 4 [file 12917_2026_5557_MOESM4_ESM.docx]

**Additional file 4**

**Table S5:** Percentage BAL fluid return relative to instilled volume per culture result

| **Culture result for *Pasteurellaceae* spp.** | **60ml or ≥100 ml** | |  | **60ml** | |  | **≥100 ml** | |
| --- | --- | --- | --- | --- | --- | --- | --- | --- |
|  | n | Median % (range) |  | n | Median % (range) |  | n | Median % (range) |
| **Total (all cultures)** | 93 | 30 (7-63) |  | 79 | 35 (14-63) |  | 14 | 23 (7-47) |
| **Culture negative** | 65 | 33 (7-63) |  | 56 | 35 (15-63) |  | 9 | 22 (7-47) |
| **Culture positive, total** | 28 | 27 (14-47) |  | 23 | 28 (14-47) |  | 5 | 25 (14-28) |
| **Culture positive, < 10 colonies** | 16 | 28 (15-47) |  | 14 | 28 (15-47) |  | 2 | 25 (22-28) |
| **Culture positive, ≥ 10 colonies** | 12 | 25 (14-40) |  | 9 | 30 (14-40) |  | 3 | 25 (14-26) |

Results are based on culture results of *Pasteurellaceae* spp. in 93 calves from nine dairy herds with enzootic pneumonia. Most calves (n=79) were given 60ml during the BAL procedure. The exceptions were 14 calves, including two calves given 100ml, and 12 calves given 120ml, due to a second instilment of 60ml after a failed return of the first 60ml instilled. In calves given 60ml twice, fluid return was calculated based on a total of 120ml.
