# Supplementary material for: Cross-sectional study of calves from Norwegian dairy herds with enzootic pneumonia: pathogen occurrence, antimicrobial resistance, culture result interpretation, and sampling site agreement
Source: BMC Vet Res. 2026 May 18;22:503. doi: 10.1186/s12917-026-05557-x (PMC13523226; doi:10.1186/s12917-026-05557-x)
Supplement: Supplementary file 5 [file 12917_2026_5557_MOESM5_ESM.docx]

**Additional file 5**

**Table S6**: Comparison of phenotypic AMR pattern of *P. multocida* isolates within sample and animal.

| **Herd** | **Animal ID** | **Health status** | **Isolates retrieved (n= 53)** | | | **Susceptibility profile** |
| --- | --- | --- | --- | --- | --- | --- |
|  |  |  | **NS (n=20)** | **NPS (n=17)** | **BAL (n=16)** |  |
| C | C8 | Diseased | 2 | 0 | 1 | All were susceptible to PEN, AMC, STX, TET, ENR, FFC and STREP. |
| B | B3 | Healthy | 0 | 2 | 0 | All were susceptible to PEN, AMC, STX, TET, ENR, FFC; and all were resistant to STREP. |
| E | E7 | Diseased | 2 | 2 | 0 |  |
| E | E10 | Diseased | 4 | 3 | 3 |  |
| F | F3 | Diseased | 4 | 4 | 4 |  |
| F | F11 | Diseased | 2 | 1 | 1 |  |
| G | G1 | Diseased | 3 | 1 | 3 |  |
| I | I10 | Healthy | 1 | 2 | 1 |  |
| I | I12 | Diseased | 2 | 2 | 3 |  |

The phenotypic AMR patterns of *P. multocida* isolates collected from NS, NPS, and BAL samples in nine animals from six herds (n=53 isolates from 23 samples). Of the 53 isolates in this analysis, one representative isolate per sampling site (n=23) was also included in other analyses in the main study. Additional isolates per sampling site (n=30) were only described in this supplementary analysis. Abbreviations: NS = nasal swabs; NPS = nasopharyngeal swabs; BAL = bronchoalveolar lavage; PEN = penicillin; AMC = amoxicillin-clavulanic acid; STX = trimethoprim + sulfa; TET = tetracycline; ENR = enrofloxacin; FFC = florfenicol; STREP = streptomycin.
