# Supplementary material for: Cross-sectional study of calves from Norwegian dairy herds with enzootic pneumonia: pathogen occurrence, antimicrobial resistance, culture result interpretation, and sampling site agreement
Source: BMC Vet Res. 2026 May 18;22:503. doi: 10.1186/s12917-026-05557-x (PMC13523226; doi:10.1186/s12917-026-05557-x)
Supplement: Supplementary file 6 [file 12917_2026_5557_MOESM6_ESM.docx]

**Additional file 6**

**Table S7:** Clinical scores of 72 healthy and 59 diseased calves from nine dairy herds with enzootic pneumonia.

| **Health status** | **Total clinical score** | **Number of calves (n=131)** | **% of total** |
| --- | --- | --- | --- |
| **Healthy (n=72)** | 0 | 7 | 5.3 |
|  | 1 | 5 | 3.8 |
|  | 2 | 19 | 14.5 |
|  | 3 | 16 | 12.2 |
|  | 4 | 12 | 9.2 |
|  | 5 | 13 | 9.9 |
| **Diseased (n= 59)** | 6 | 7 | 5.3 |
|  | 7 | 12 | 9.2 |
|  | 8 | 15 | 11.5 |
|  | 9 | 5 | 3.8 |
|  | 10 | 9 | 6.9 |
|  | 11 | 5 | 3.8 |
|  | 12 | 0 | 0 |
|  | 13 | 4 | 3.1 |
|  | 14 | 1 | 0.8 |
|  | 15 | 0 | 0 |
|  | 16 | 0 | 0 |
|  | 17 | 0 | 0 |
|  | 18 | 1 | 0.8 |

An overall clinical score (CS; range 0-18) was used to classify calves as healthy (CS ≤ 5) or diseased (CS > 5).
