# Supplementary material for: Cross-sectional study of calves from Norwegian dairy herds with enzootic pneumonia: pathogen occurrence, antimicrobial resistance, culture result interpretation, and sampling site agreement
Source: BMC Vet Res. 2026 May 18;22:503. doi: 10.1186/s12917-026-05557-x (PMC13523226; doi:10.1186/s12917-026-05557-x)
Supplement: Supplementary file 7 [file 12917_2026_5557_MOESM7_ESM.docx]

**Additional file 7**

**Table S8:** Distribution per herd of *Pasteurellaceae* spp. among healthy and diseased calves.

| Herd | Culture result detected^1^ | Percentages (n) of each culture result | | | | | | | | | | | | | | | | | | | |
| --- | --- | --- | --- | --- | --- | --- | --- | --- | --- | --- | --- | --- | --- | --- | --- | --- | --- | --- | --- | --- | --- |
|  |  | **Calf^2^**  *N = 131; 72 H, 59 D* | | |  | **NS**  *N = 131; 72 H, 59 D* | | |  | **NPS**  *N = 131; 72 H, 59 D* | | |  | **BAL**  *N = 113; 66 H, 47 D* | | |  | **Enriched BAL^3^**  *N = 62; 36 H, 26 D* | | |  |
|  |  | **Total** | **H** | **D** |  | **Total** | **H** | **D** |  | **Total** | **H** | **D** |  | **Total** | **H** | **D** |  | **Total** | **H** | **D** |  |
| A | *P. multocida* | 85 %  (17/20) | 78 %  (7/9) | 91 %  (10/11) |  | 75 %  (15/20) | 78 % (7/9) | 73 % (8/11) |  | 80 % (16/20) | 67 % (6/9) | 91 % (10/11) |  | 60 % (6/10) | 57 % (4/7) | 67 % (2/3) |  | N/A | N/A | N/A |  |
|  | *M. haemolytica* | 25 % (5/20) | 33 % (3/9) | 18 % (2/11) |  | 20 % (4/20) | 22 % (2/9) | 18 % (2/11) |  | 15 % (3/20) | 11 % (1/9) | 18 % (2/11) |  | 10 % (1/10) | 14 % (1/7) | 0 % (0/3) |  | N/A | N/A | N/A |  |
|  | No *Pasteurellaceae* | 15 % (3/20) | 22 % (2/9) | 9 % (1/11) |  | 20 % (4/20) | 22 % (2/9) | 18 % (2/11) |  | 20 % (4/20) | 33 % (3/9) | 9 % (1/11) |  | 30 % (3/10) | 29 % (2/7) | 33 % (1/3) |  | N/A | N/A | N/A |  |
| B^4^ | *P. multocida* | 55 % (11/20) | 50 % (9/18) | 100 % (2/2) |  | 25 % (5/20) | 17 % (3/18) | 100 % (2/2) |  | 25 % (5/20) | 22 % (4/18) | 50 % (1/2) |  | 18 % (3/17) | 20 % (3/15) | 0 % (0/2) |  | 40 % (4/10) | 25 % (2/8) | 100 % (2/2) |  |
|  | *M. haemolytica* | 60 % (12/20) | 56 % (10/18) | 100 % (2/2) |  | 50 % (10/20) | 44 % (8/18) | 100 % (2/2) |  | 30 % (6/20) | 28 % (5/18) | 50 % (1/2) |  | 0 % (0/17) | 0 % (0/15) | 0 % (0/2) |  | 10 % (1/10) | 13 % (1/8) | 0 % (0/2) |  |
|  | No *Pasteurellaceae* | 20 % (4/20) | 22 % (4/18) | 0 %  (0/2) |  | 40 % (8/20) | 44 % (8/18) | 0 % (0/2) |  | 55 % (11/20) | 56 % (10/18) | 50 % (1/2) |  | 82 % (14/17) | 80 % (12/15) | 100 % (2/2) |  | 50 % (5/10) | 63 % (5/8) | 0 % (0/2) |  |
| C^4^ | *P. multocida* | 33 % (5/15) | 30 % (3/10) | 40 % (2/5) |  | 13 % (2/15) | 10 % (1/10) | 20 % (1/5) |  | 7 % (1/15) | 10 % (1/10) | 0 % (0/5) |  | 15 % (2/13) | 11 % (1/9) | 25 % (1/4) |  | 30 % (3/10) | 25 % (2/8) | 50 % (1/2) |  |
|  | *M. haemolytica* | 73 % (11/15) | 70 % (7/10) | 80 % (4/5) |  | 73 % (11/15) | 70 % (7/10) | 80 % (4/5) |  | 47 % (7/15) | 40 % (4/10) | 60 % (3/5) |  | 8 % (1/13) | 0 % (0/9) | 25 % (1/4) |  | 20 % (2/10) | 25 % (2/8) | 0 % (0/2) |  |
|  | No *Pasteurellaceae* | 27 % (4/15) | 30 % (3/10) | 20 % (1/5) |  | 27 % (4/15) | 30 % (3/10) | 20 % (1/5) |  | 47 % (7/15) | 50 % (5/10) | 40 % (2/5) |  | 77 % (10/13) | 89 % (8/9) | 50 % (2/4) |  | 50 % (5/10) | 50 % (4/8) | 50 % (1/2) |  |
| D | *M. haemolytica* | 47 % (8/17) | 60 % (3/5) | 42 % (5/12) |  | 35 % (6/17) | 40 % (2/5) | 33 % (4/12) |  | 24 % (4/17) | 20 % (1/5) | 25 % (3/12) |  | 6 % (1/17) | 0 % (0/5) | 8 % (1/12) |  | 14 % (2/14) | 20 % (1/5) | 11 % (1/9) |  |
|  | No *Pasteurellaceae* | 53 % (9/17) | 40 % (2/5) | 58 % (7/12) |  | 65 % (11/17) | 60 % (3/5) | 67 % (8/12) |  | 76 % (13/17) | 80 % (4/5) | 75 % (9/12) |  | 94 % (16/17) | 100 % (5/5) | 92 % (11/12) |  | 86 % (12/14) | 80 % (4/5) | 89 % (8/9) |  |
| E | *P. multocida* | 100 % (11/11) | 100 % (4/4) | 100 % (7/7) |  | 91 % (10/11) | 100 % (4/4) | 86 % (6/7) |  | 100 % (11/11) | 100 % (4/4) | 100 % (7/7) |  | 10 % (1/10) | 0 % (0/4) | 17 % (1/6) |  | 33 % (3/9) | 25 % (1/4) | 40 % (2/5) |  |
|  | *M. haemolytica* | 55 % (6/11) | 75 % (3/4) | 43 % (3/7) |  | 55 % (6/11) | 75 % (3/4) | 43 % (3/7) |  | 45 % (5/11) | 75 % (3/4) | 29 % (2/7) |  | 0 % (0/10) | 0 % (0/4) | 0 % (0/6) |  | 0 % (0/9) | 0 % (0/4) | 0 % (0/5) |  |
|  | *H. somni* | 27 % (3/11) | 0 % (0/4) | 43 % (3/7) |  | 18 % (2/11) | 0 % (0/4) | 29 % (2/7) |  | 18 % (2/11) | 0 % (0/4) | 29 % (2/7) |  | 0 % (0/10) | 0 % (0/4) | 0 % (0/6) |  | 0 % (0/9) | 0 % (0/4) | 0 % (0/5) |  |
|  | No *Pasteurellaceae* | 0 % (0/11) | 0 % (0/4) | 0 %  (0/7) |  | 0 % (0/11) | 0 % (0/4) | 0 % (0/7) |  | 0 % (0/11) | 0 % (0/4) | 0 % (0/7) |  | 90 % (9/10) | 100 % (4/4) | 83 % (5/6) |  | 67 % (6/9) | 75 % (3/4) | 60 % (3/5) |  |
| F | *P. multocida* | 100 % (12/12) | 100 % (5/5) | 100 % (7/7) |  | 100 % (12/12) | 100 % (5/5) | 100 % (7/7) |  | 100 % (12/12) | 100 % (5/5) | 100 % (7/7) |  | 92 % (11/12) | 80 % (4/5) | 100 % (7/7) |  | N/A | N/A | N/A |  |
|  | *M. haemolytica* | 33 % (4/12) | 20 % (1/5) | 43 % (3/7) |  | 33 % (4/12) | 20 % (1/5) | 43 % (3/7) |  | 17 % (2/12) | 20 % (1/5) | 14 % (1/7) |  | 8 % (1/12) | 20 % (1/5) | 0 % (0/7) |  | N/A | N/A | N/A |  |
|  | *H. somni* | 8 % (1/12) | 20 % (1/5) | 0 %  (0/7) |  | 0 % (0/12) | 0 % (0/5) | 0 % (0/7) |  | 0 % (0/12) | 0 % (0/5) | 0 % (0/7) |  | 8 % (1/12) | 20 % (1/5) | 0 % (0/7) |  | N/A | N/A | N/A |  |
|  | No *Pasteurellaceae* | 0 % (0/12) | 0 % (0/5) | 0 %  (0/7) |  | 0 % (0/12) | 0 % (0/5) | 0 % (0/7) |  | 0 % (0/12) | 0 % (0/5) | 0 % (0/7) |  | 8 % (1/12) | 20 % (1/5) | 0 % (0/7) |  | N/A | N/A | N/A |  |
| G | *P. multocida* | 67 % (8/12) | 67 % (4/6) | 67 % (4/6) |  | 50 % (6/12) | 50 % (3/6) | 50 % (3/6) |  | 50 % (6/12) | 50 % (3/6) | 50 % (3/6) |  | 30 % (3/10) | 33 % (2/6) | 25 % (1/4) |  | 20 % (1/5) | 0 % (0/2) | 33 % (1/3) |  |
|  | *M. haemolytica* | 75 % (9/12) | 67 % (4/6) | 83 % (5/6) |  | 58 % (7/12) | 33 % (2/6) | 83 % (5/6) |  | 67 % (8/12) | 67 % (4/6) | 67 % (4/6) |  | 20 % (2/10) | 33 % (2/6) | 0 % (0/4) |  | 20 % (1/5) | 0 % (0/2) | 33 % (1/3) |  |
|  | No *Pasteurellaceae* | 0 % (0/12) | 0 % (0/6) | 0 %  (0/6) |  | 8 % (1/12) | 17 % (1/6) | 0 % (0/6) |  | 8 % (1/12) | 0 % (0/6) | 17 % (1/6) |  | 60 % (6/10) | 50 % (3/6) | 75 % (3/4) |  | 80 % (4/5) | 100 % (2/2) | 67 % (2/3) |  |
| H^4^ | *P. multocida* | 25 % (3/12) | 13 % (1/8) | 50 % (2/4) |  | 17 % (2/12) | 13 % (1/8) | 25 % (1/4) |  | 8 % (1/12) | 13 % (1/8) | 0 % (0/4) |  | 8 % (1/12) | 0 % (0/8) | 25 % (1/4) |  | 20 % (1/5) | 25 % (1/4) | 0 % (0/1) |  |
|  | *M. haemolytica* | 75 % (9/12) | 75 % (6/8) | 75 % (3/4) |  | 58 % (7/12) | 75 % (6/8) | 25 % (1/4) |  | 67 % (8/12) | 63 % (5/8) | 75 % (3/4) |  | 25 % (3/12) | 25 % (2/8) | 25 % (1/4) |  | 0 % (0/5) | 0 % (0/4) | 0 % (0/1) |  |
|  | No *Pasteurellaceae* | 17 % (2/12) | 25 % (2/8) | 0 %  (0/4) |  | 33 % (4/12) | 25 % (2/8) | 50 % (2/4) |  | 33 % (4/12) | 38 % (3/8) | 25 % (1/4) |  | 67 % (8/12) | 75 % (6/8) | 50 % (2/4) |  | 80 % (4/5) | 75 % (3/4) | 100 % (1/1) |  |
| I | *P. multocida* | 92 % (11/12) | 100 % (7/7) | 80 % (4/5) |  | 83 % (10/12) | 86 % (6/7) | 80 % (4/5) |  | 58 % (7/12) | 43 % (3/7) | 80 % (4/5) |  | 25 % (3/12) | 29 % (2/7) | 20 % (1/5) |  | 67 % (6/9) | 60 % (3/5) | 75 % (3/4) |  |
|  | *M. haemolytica* | 67 % (8/12) | 86 % (6/7) | 40 % (2/5) |  | 42 % (5/12) | 57 % (4/7) | 20 % (1/5) |  | 42 % (5/12) | 57 % (4/7) | 20 % (1/5) |  | 0 % (0/12) | 0 % (0/7) | 0 % (0/5) |  | 0 % (0/9) | 0 % (0/5) | 0 % (0/4) |  |
|  | No *Pasteurellaceae* | 0 % (0/12) | 0 % (0/7) | 0 %  (0/5) |  | 17 % (2/12) | 14 % (1/7) | 20 % (1/5) |  | 8 % (1/12) | 14 % (1/7) | 0 % (0/5) |  | 75 % (9/12) | 71 % (5/7) | 80 % (4/5) |  | 33 % (3/9) | 40 % (2/5) | 25 % (1/4) |  |

Results are based on 72 healthy and 59 diseased calves from nine dairy herds with enzootic pneumonia. Abbreviations: NS = nasal swab; NPS = nasopharyngeal swab; BAL = bronchoalveolar lavage; H = healthy; D = diseased.

^1^*Pasteurellaceae* spp. were only listed in a herd in this table if ≥ 1 calf in the herd was positive for that species.

^2^A calf was classified as positive if ≥ 1 sampling site (NS, NPS, BAL, including enriched BAL samples) was positive.

^3^Enriched BAL = BAL fluid enriched with BHI broth following bacteria negative culture from the corresponding non-enriched BAL fluid, providing additional isolates from the lower airways.

^4^Herd with predominantly healthy calves (herd B, C, and H).
