# Supplementary material for: Cross-sectional study of calves from Norwegian dairy herds with enzootic pneumonia: pathogen occurrence, antimicrobial resistance, culture result interpretation, and sampling site agreement
Source: BMC Vet Res. 2026 May 18;22:503. doi: 10.1186/s12917-026-05557-x (PMC13523226; doi:10.1186/s12917-026-05557-x)
Supplement: Supplementary file 8 [file 12917_2026_5557_MOESM8_ESM.docx]

**Additional file 8**

**Table S9:** Univariable analyses describing the associations between health status and potential predictors.

| Bacterial predictor variables^1^ | Level (n)^3^ | β^4^ | OR^4^ | SE^4^ | 95% CI^4^ | *P^4^* | N^4^ |
| --- | --- | --- | --- | --- | --- | --- | --- |
| *P. multocida* detected*,* NS | Negative (69) | - | 1.0 | - | - | - | - |
|  | Positive (62) | 0.49 | 1.64 | 0.71 | 0.70-3.81 | 0.25 | 131 |
| *P. multocida* detected, NPS | Negative (72) | - | 1.0 | - | - | - | - |
|  | Positive (59) | 0.71 | 2.04 | 0.9 | 0.86-4.84 | 0.10 | 131 |
| *P. multocida* dominant, NPS | Negative (80) | - | 1.0 | - | - | - | - |
|  | Positive (51) | 0.45 | 1.57 | 0.68 | 0.67-3.66 | 0.30 | 131 |
| *M. haemolytica* dominant, NS | Negative (104) | - | 1.0 | - | - | - | - |
|  | Positive (27) | -0.79 | 0.45 | 0.23 | 0.17-1.23 | 0.12 | 131 |
| *P. multocida* dominant, abundant, NS | Negative (84) | - | 1.0 | - | - | - | - |
|  | Positive (47) | 0.42 | 1.53 | 0.66 | 0.66-3.55 | 0.33 | 131 |
| *P. multocida* dominant, abundant, BAL | Negative (98) | - | 1.0 | - | - | - | - |
|  | Positive (15) | 1.50 | 4.46 | 3.2 | 1.09-18.17 | 0.04 | 113 |
| **Other predictor variables^1,2^** |  |  |  |  |  |  |  |
| Age^cont^ | - | -0.00 | 1.0 | 0.00 | 0.99-1.00 | 0.31 | 131 |
| Age | < 60 days (62) | - | 1.0 | - | - | - | - |
|  | ≥ 60 days (69) | 0.01 | 1.01 | 0.40 | 0.46-2.21 | 0.98 | 131 |
| Breed | Norwegian Red (109) | - | 1.0 | - | - | - | - |
|  | Other breeds (22) | -0.46 | 0.63 | 0.39 | 0.19-2.10 | 0.45 | 131 |
| Gender | Female (69) | - | 1.0 | - | - | - | - |
|  | Male (62) | 0.93 | 2.52 | 0.99 | 1.17-5.46 | 0.02 | 131 |

Associations between health status (0 = healthy, baseline; 1 = diseased) and predictor variables in 131 calves (72 healthy and 59 diseased) from nine dairy herds with enzootic pneumonia. Herd was included as a random effect. Abbreviations: NS = nasal swab; NPS = nasopharyngeal swab; BAL = bronchoalveolar lavage.

^1^Categorical variables: All bacterial predictor variables; age; breed; and gender

^2^Continuous variables: Age^cont^

^3^Baselines of predictor variables: Bacterial predictor variables = negative; Age = < 60 days; Breed = Norwegian Red; Gender = female

^4^Model output: β = beta coefficient; OR = odds ratio; SE = standard error; CI = confidence interval; P = p-value; N = sample size.
