# Supplementary material for: Cross-sectional study of calves from Norwegian dairy herds with enzootic pneumonia: pathogen occurrence, antimicrobial resistance, culture result interpretation, and sampling site agreement
Source: BMC Vet Res. 2026 May 18;22:503. doi: 10.1186/s12917-026-05557-x (PMC13523226; doi:10.1186/s12917-026-05557-x)
Supplement: Supplementary file 9 [file 12917_2026_5557_MOESM9_ESM.docx]

**Additional file 9**

**Table S10:** Cross-tabulation of *P. multocida* culture results from upper airway sites vs. BAL by health status

|  |  |  | **Culture result for *P. multocida* in BAL/upper airway site** | | | | | | | | | | | | | | |
| --- | --- | --- | --- | --- | --- | --- | --- | --- | --- | --- | --- | --- | --- | --- | --- | --- | --- |
|  |  |  | **Detection in BAL** | | | |  | **Dominant in BAL** | | | |  | **Dominant, abundant in BAL** | | | | |
| **Calf health status** | **Upper airway site** | **Culture result for P. multocida** | +/+ | +/- | -/+ | -/- |  | +/+ | +/- | -/+ | -/- |  | +/+ | +/- | -/+ | -/- |  |
| **Healthy**  **(n=66)** | NS | Detected | 11 | 5 | 17 | 33 |  | 11 | 2 | 17 | 36 |  | 5 | 0 | 23 | 38 |  |
|  |  | Dominant | 10 | 6 | 13 | 37 |  | 10 | 3 | 13 | 40 |  | 5 | 0 | 18 | 43 |  |
|  |  | *- Pure^1^* | *2* | *14* | *2* | *48* |  | *2* | *11* | *2* | *51* |  | *0* | *5* | *4* | *57* |  |
|  |  | Dominant+ abundant | 9 | 7 | 12 | 38 |  | 9 | 4 | 12 | 41 |  | 5 | 0 | 16 | 45 |  |
|  | NPS | Detected | 11 | 5 | 14 | 36 |  | 10 | 3 | 15 | 38 |  | 5 | 0 | 20 | 41 |  |
|  |  | Dominant | 8 | 8 | 14 | 36 |  | 8 | 5 | 14 | 39 |  | 4 | 1 | 18 | 43 |  |
|  |  | *- Pure^1^* | *3* | *13* | *6* | *44* |  | *3* | *10* | *6* | *47* |  | *0* | *5* | *9* | *52* |  |
|  |  | Dominant+ abundant | 3 | 13 | 4 | 46 |  | 3 | 10 | 4 | 49 |  | 1 | 4 | 6 | 55 |  |
| **Diseased**  **(n= 47)** | NS | Detected | 13 | 1 | 12 | 21 |  | 12 | 0 | 13 | 22 |  | 10 | 0 | 15 | 22 |  |
|  |  | Dominant | 12 | 2 | 10 | 23 |  | 12 | 0 | 10 | 25 |  | 10 | 0 | 12 | 25 |  |
|  |  | *- Pure^1^* | *0* | *14* | *2* | *31* |  | *0* | *12* | *2* | *33* |  | *0* | *10* | *2* | *35* |  |
|  |  | Dominant+ abundant | 12 | 2 | 10 | 23 |  | 12 | 0 | 10 | 25 |  | 10 | 0 | 12 | 25 |  |
|  | NPS | Detected | 12 | 2 | 11 | 22 |  | 12 | 0 | 11 | 24 |  | 10 | 0 | 13 | 24 |  |
|  |  | Dominant | 8 | 6 | 11 | 22 |  | 8 | 4 | 11 | 24 |  | 6 | 4 | 13 | 24 |  |
|  |  | *- Pure^1^* | *6* | *8* | *7* | *26* |  | *6* | *6* | *7* | *28* |  | *5* | *5* | *8* | *29* |  |
|  |  | Dominant+ abundant | 8 | 6 | 11 | 22 |  | 8 | 4 | 11 | 24 |  | 6 | 4 | 13 | 24 |  |

Culture results (BAL/upper airway site) are based on 66 healthy and 47 diseased calves with paired sampling sites from nine dairy herds with enzootic pneumonia. No statistical analyses were done for healthy and diseased calves separately due to insufficient sample size at the group level. Abbreviations: NS = nasal swab; NPS = nasopharyngeal swab; BAL = bronchoalveolar lavage.

^1^Pure cultures were also part of the “dominant” cultures category.
